# Biosynthetic Pathways of Hormones in Plants

**DOI:** 10.3390/metabo13080884

**Published:** 2023-07-25

**Authors:** Andrzej Bajguz, Alicja Piotrowska-Niczyporuk

**Affiliations:** Department of Biology and Plant Ecology, Faculty of Biology, University of Bialystok, Ciolkowskiego 1J, 15-245 Bialystok, Poland; alicjap@uwb.edu.pl

**Keywords:** abscisic acid, auxins, biosynthesis, brassinosteroids, cytokinins, ethylene, gibberellins, jasmonic acid, melatonin, polyamines, salicylic acid, strigolactones

## Abstract

Phytohormones exhibit a wide range of chemical structures, though they primarily originate from three key metabolic precursors: amino acids, isoprenoids, and lipids. Specific amino acids, such as tryptophan, methionine, phenylalanine, and arginine, contribute to the production of various phytohormones, including auxins, melatonin, ethylene, salicylic acid, and polyamines. Isoprenoids are the foundation of five phytohormone categories: cytokinins, brassinosteroids, gibberellins, abscisic acid, and strigolactones. Furthermore, lipids, i.e., α-linolenic acid, function as a precursor for jasmonic acid. The biosynthesis routes of these different plant hormones are intricately complex. Understanding of these processes can greatly enhance our knowledge of how these hormones regulate plant growth, development, and physiology. This review focuses on detailing the biosynthetic pathways of phytohormones.

## 1. Introduction

**Figure 1:**
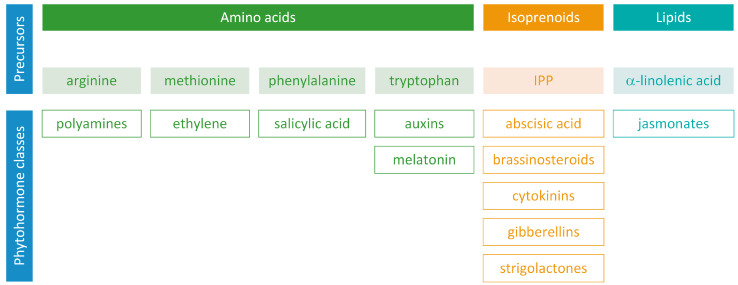
Precursors of phytohormone biosynthesis.

Phytohormones, which are also known as plant hormones, are small, naturally occurring organic compounds that significantly influence the growth, development, defense, productivity, and physiological mechanisms of plants. They also orchestrate various cellular activities within the plant. Even at minimal concentrations, they are operative in plant cells, tissues, and organs. They are found in all vascular plants and a substantial number of non-vascular species (Table 1). From the initial discovery of auxin to the most recent unearthing of strigolactones (SLs), 12 groups of phytohormones—auxins, cytokinins (CKs), gibberellins (GAs), abscisic acid (ABA), ethylene, brassinosteroids (BRs), salicylic acid (SA), jasmonates, polyamines (PAs), melatonin, SLs, and peptide hormones—have been identified in numerous plant species. The various chemical structures of phytohormones are pivotal for their diverse biological functions and biosynthesis (Figure 1):

- auxins and melatonin are indole derivatives;
- ABA is a sesquiterpene;
- ethylene is the simplest alkene;
- CKs are adenine analogues;
- GAs are tetracyclic diterpenoid acids;
- BRs are polyhydroxysteroids;
- jasmonates are derived from fatty acids;
- PAs are aliphatic nitrogenous bases;
- SA is a phenolic organic acid;
- SLs are terpenoid lactones [1,2].

In addition, peptide hormones regulate many developmental and defense processes, such as meristem maintenance, xylem and phloem differentiation, stomata patterning, pollination, embryo and endosperm development, cell division, nodulation, and systematic response [3]. They are divided into secreted and non-secreted types. Secreted peptide hormones are further divided into post-translationally modified peptides and cysteine-rich peptides. Peptide hormones are synthesized as larger precursor molecules, which are then cleaved to produce active peptides, e.g., CLAVATA3 (CLV3)/Embryo Surrounding Region-Related (CLE), Phytosulfokine (PSK), Plant Peptide-Containing Sulfated Tyrosine (PSY) peptides belong to post-translationally modified peptides; Rapid Alkalinization Factor (RALF) peptides belong to cysteine-rich peptides; Plant Elicitor Peptides (PEP)—to non-secreted peptides. Peptide hormones are also generated by plant pathogens, symbionts, and microbes that interact with plants. They are crucial in establishing a molecular interface that allows them to co-exist with the host plant. These organisms produce effectors that mimic peptide phytohormones and other effectors of pathogens and symbionts. Plant receptors recognize these effectors, which primarily regulate growth rather than defense responses. However, the origin of non-plant peptide phytohormones is still a subject of controversy [3,4,5,6]. Although peptide hormones are considered a legitimate group of plant hormones, their biosynthesis is not covered by the scope of this article because their biosynthesis shares all of the typical characteristics of peptide biosynthesis.

**Table 1:** The localization of hormones in plants [7,8].

Phytohormone Class	Occurrence and Site of Biosynthesis
Abscisic acid	Roots, mature leaves, particularly in response to water stress, seeds
Auxins	Leaf primordia, young leaves, developing seeds
Brassinosteroids	Leaves, shoots, roots, fruits, seeds, pollen
Cytokinins	Root tips, developing seeds, leaves, stem, flowers, siliques, fruits, shoot meristem
Ethylene	Tissues undergoing ripening (fruits), roots, shoots, particularly in response to stress
Gibberellins	Young tissues of the shoot, developing seeds
Jasmonates	Leaves, roots
Melatonin	Leaves, stems, roots, fruits, seeds
Polyamines	Most tissues, particularly in response to stress, in tissues undergoing senescence or ripening
Salicylic acid	Leaves, particularly in response to pathogenic attack
Strigolactones	Roots, shoots

As such, plants host diverse phytohormone pathways. Significant advancements have been made in the study of phytohormone biology and synthesis over the past decade. An assortment of new tools and methods has been developed, resulting in the discovery of phytohormone substrates, intermediates, and final products. The biosynthetic pathways of plant hormones have been elucidated, and extensive research has been carried out into the genes found in the plant genome, encoding the enzymes that catalyze the various stages of phytohormone synthesis [1,2].

This review aims to outline the biosynthetic pathways of non-peptide plant hormones (Figure 1 and Figure S1).

## 2. Polyamines

**Figure 2:**
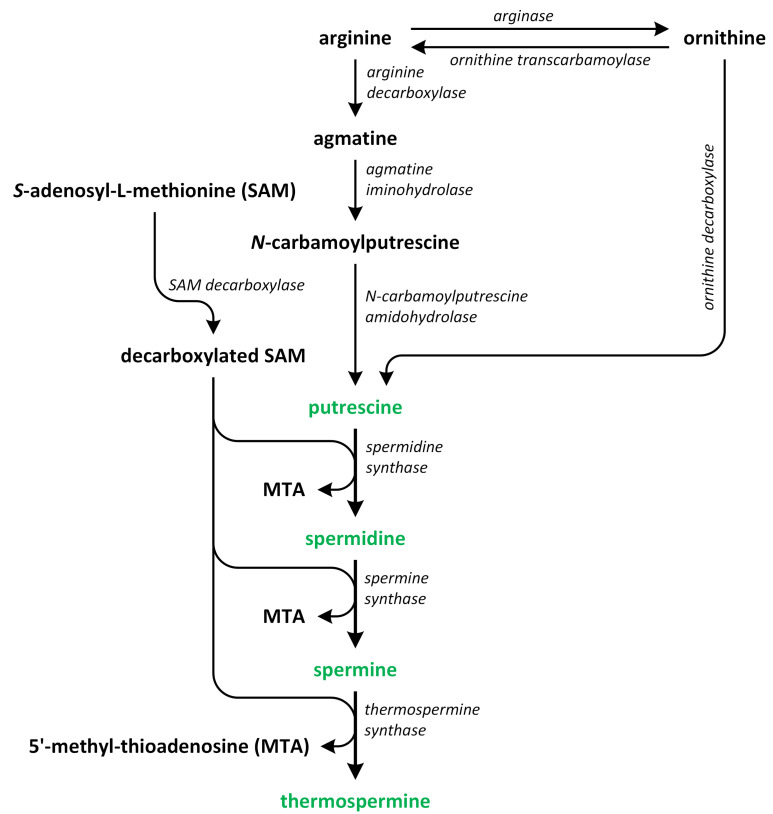
The biosynthetic pathways of polyamines (phytohormones are green).

Polyamines (PAs) play a crucial role in plant growth, metabolism, and development. Structurally, these compounds are organic polycationic alkylamines that contain two or more amino groups. PAs such as putrescine (Put), spermidine (Spd), and spermine (Spm) are the most well-studied and recognized PAs found in plants. These three PAs regulate various physiological activities, such as photosynthesis, flower generation, embryogenesis, and organogenesis. They are also accountable for maintaining the stability of nucleic acids, various protein molecules, and the membrane structure. Additionally, they play a substantial role in enhancing the tolerance of many plants to the presence of a variety of abiotic and biotic stress factors, thereby impacting crop yield. Putrescine is a key compound involved in the biosynthesis of PAs. It serves as a common intermediate in the creation of Spd, Spm, and thermospermine. The biosynthesis pathways of Put have been identified in many plants (Figure 2) [9,10,11,12].

In the first pathway, arginine (Arg) is decarboxylated through the action of enzyme argi-nine decarboxylase (ADC). This enzyme acts on the carbon at position 8. In this reaction, agmatine (Agm) and CO_2_ (the ADC pathway) are produced. Subsequently, Agm is used to synthesize *N*-carbamoyl putrescine (NCPA) and ammonia (NH_3_), which occurs following the removal of nitrogen. In the succeeding reaction, NCPA amidohydrolase hydrolyzes NCPA, removing its carbamoyl group to produce NH_3_, CO_2_, and the first PA—Put. This process is the most prevalent pathway for Put synthesis in plants [13,14].

In the second pathway of Put biosynthesis, ornithine (Orn) is formed from Arg in the reaction catalyzed by arginase [15]. Next, the enzyme ornithine decarboxylase (ODC) removes the carboxyl group bonded to C-1 of Orn to form products such as Put and CO_2_ [13]. On the other hand, the ODC gene that encodes this enzyme has not been identified in *Arabidopsis thaliana* and other plants from the Brassicaceae family. Therefore, there is a high possibility that the ornithine pathway, which is known also as ODC pathway, is not important for normal plant growth and development in relation to the ADC pathway [16,17]. However, other authors speculate that the ODC pathway is mainly involved in regulation of plant growth, development, differentiation of organs, and reproduction, while the ADC pathway is activated in plants that grow under unfavorable environmental conditions. These two pathways are commonly found in plants [10,16,17,18,19,20].

The third pathway is the least known route of Put formation in plants. Other PAs, such as Spd and Spm, are biosynthesized from Put. Decarboxylated *S*-adenosyl-L-methionine (dcSAM) is a source of aminopropyl residues, which are the indispensable intermediates involved in the polyamine biosynthetic pathway [10,21]. For dcSAM production, S-adenosyl-L-methionine (SAM) is decarboxylated through the activity of the enzyme S-adenosyl-L-methionine decarboxylases (SAMDC) [22]. Aminopropyl groups are added to the Put molecule in a reaction catalyzed by spermidine synthase (SPDS), and Spd is produced. In *A. thaliana*, two genes, i.e., *SPDS1* (*At1g23820*) and *SPDS2* (*At1g70310*) that encode SPDS, as well as four *SAMDC1-4* genes, i.e., *At3g02470*, *At3g25570*, *At5g15959*, and *At5g18930*, that encode SAMDC, which are essential for plant embryogenesis, have been identified [23]. The last step of the biosynthesis of PAs, i.e., the transfer of aminopropyl groups to propylamine acceptor Spd, leads to the formation of Spm. In this reaction, this Spm is formed via the action of spermine synthase (SPMS) [18,24,25,26]. The biosynthesis of PAs in plants is closely connected to the biosynthetic pathways of other plant hormones. For example, SAM, which is present in the PA biosynthetic pathway, plays also a key role as a precursor involved in ethylene biosynthesis, and past studies showed that the production of PAs can compete with ethylene [27,28].

## 3. Ethylene

The volatile phytohormone ethylene plays a key role in controlling various developmental and physiological activities, including seed dormancy and germination, growth of vegetation, flowering, maturation of climacteric fruit, and aging. Moreover, ethylene is seen as a critical component that protects plants from both biological and non-biological stressors. Being a gaseous plant hormone, ethylene has the ability to easily spread from its creation sites without needing any biochemical alterations or metabolic processes, allowing it to be sensed promptly [29].

Ethylene production is intricately controlled by internal cues throughout its development. Additionally, ethylene production can be swayed by environmental triggers, including biological triggers, like an onslaught of pathogens, and non-biological triggers, like injury, low oxygen levels, exposure to ozone, cold temperatures, or freezing conditions [30].

**Figure 3:**
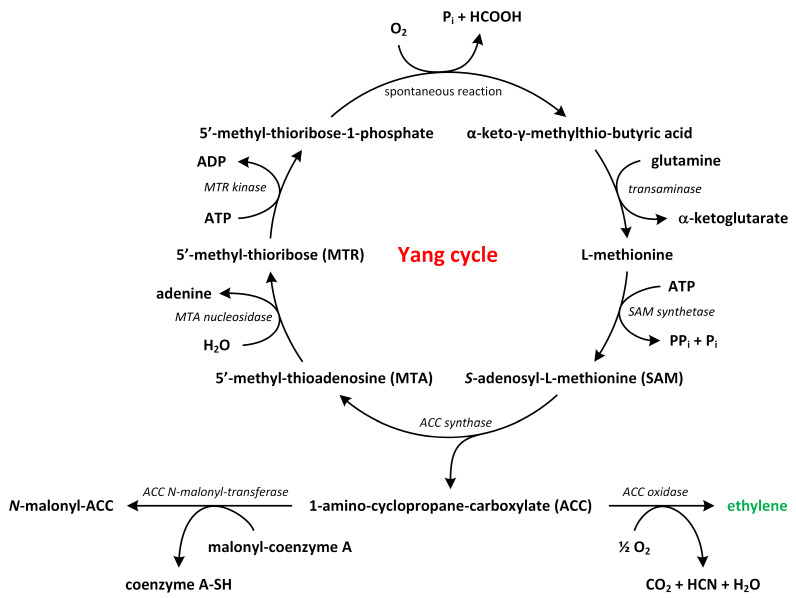
The biosynthetic pathways of ethylene (phytohormone is green).

The biosynthesis of ethylene is a relatively straightforward process that involves two key enzymatic reactions (Figure 3).

Methionine, which is the precursor of ethylene, is converted to S-adenosylmethionine (SAM) by the enzyme S-adenosylmethionine synthetase. Around 80% of cellular methionine is directed towards SAM synthesis, which is involved in various pathways, including polyamines (PAs) and ethylene biosynthesis [31,32,33]. SAM also participates in methylation reactions that modify lipids, proteins, and nucleic acids [30]. Next, SAM is transformed into 1-aminocyclopropane-1-carboxylic acid (ACC) and 5′-methyl-thioadenosine (MTA) by the enzyme ACC synthase (ACS) [34,35,36]. This conversion represents the rate-limiting step in the ethylene biosynthetic pathway, as the expression of *ACS* genes is tightly regulated by environmental factors. ACS is present at low levels under normal conditions, though its activity increases in response to biotic or abiotic stress, suggesting tight control over ethylene production [37,38].

In the second step, ACC is further converted into ethylene and other by-products, such as CO_2_ and cyanide, by the enzyme ACC oxidase (ACO) (Figure 3). In *A. thaliana*, ACO proteins are encoded by five genes (*ACO1*–*5*) and belong to a superfamily of oxygenases/oxidases. The toxic cyanide by-product is rapidly detoxified through its conversion into β-cyanoalanine by β-cyanoalanine synthase [39]. Alternatively, ACC can be conjugated to malonyl-ACC, and this process regulates the availability of ACC for conversion to ethylene. By controlling the ratio of malonyl-ACC to ACC, plants can modulate ethylene biosynthesis [29,40,41].

## 4. Salicylic Acid

Salicylic acid (SA) participates in plant growth and development. In heat-generating plants, it initiates the process of heat production by triggering an alternative respiration pathway, leading to the release of foul-smelling compounds that lure pollinators. In terms of plant defense, SA acts as a messenger, managing the expression of plant pathogenesis-related genes and fostering disease resistance. Additionally, it helps to orchestrate plant reactions to diverse types of non-biological stress factors, like extreme temperatures, saline conditions, and oxidative stress. SA also has a role in controlling a range of other activities in plants, including photosynthesis, respiration, growth of vegetation, seed germination, flowering, and aging [42,43,44].

**Figure 4:**
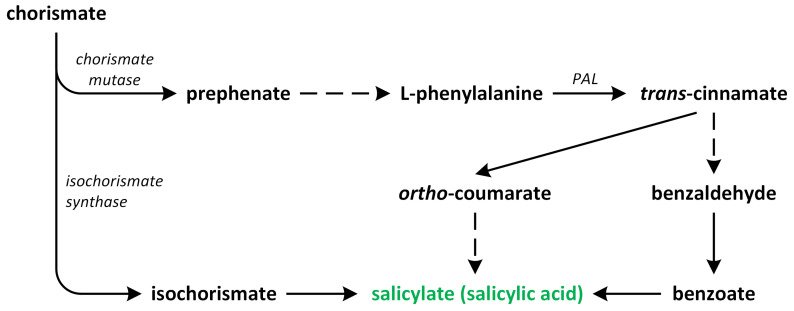
The biosynthetic pathways of salicylic acid (phytohormone is green).

In plants, SA occurs via two pathways: (i) the isochorismate synthase (ICS) (major fraction) and (ii) the phenylalanine ammonia-lyase (PAL) pathways (Figure 4). Both biosynthetic pathways start in chorismate plastids and vary between plant species [42]. Although plants employ both pathways simultaneously, the ICS pathway is the primary contributor, accounting for approximately 95% of SA synthesis. Chorismate is a primary metabolic precursor of both pathways [45]. This compound is the end product of the shikimate pathway, which is initiated by erythrose-4-phosphate and phosphoenolpyruvate [46,47].

The ICS pathway was first discovered in bacteria. Later, its presence was confirmed in vascular plants [48,49]. In the ICS pathway, the first identified step is the conversion of chorismate into its isomer, which is known as isochorismate, via the reaction catalyzed by ICS or its homologous. These enzymes are common to both bacteria and plants. *A. thaliana* and soybean (*Glycine max*) possess two genes that encode ICS, while rice (*Oryza sativa*) has only one gene that encodes this enzyme. Although the activities of ICS enzymes may vary between plant species, their primary structures are almost conserved [47]. Isochorismate is, indeed, produced in plastids and subsequently transported to the cytosol. The transportation of isochorismate from plastids to the cytosol is facilitated by the Enhanced Disease Susceptibility 5 (EDS5) protein, which functions as a MATE transporter [48]. Once it is present in the cytosol, isochorismate undergoes conjugation with L-glutamate and is converted into isochorismate-9-glutamate (ICS-Glu) through the action of a cytosolic amidotransferase. In the last step, the spontaneous decomposition of ICS-Glu leads to accumulation of the following products: SA and 2-hydroxy-acryloyl-*N*-glutamate. The activity of the cytosolic amidotransferase, which converts isochorismate into isochorismate-9-glutamate (ICS-Glu), is, indeed, inhibited by salicylic acid (SA), which acts as a negative feedback regulation mechanism. This feedback inhibition by SA helps to regulate the biosynthesis of SA and control its levels within the plant [42]. In *A. thaliana*, the ICS pathway plays a significant role in the accumulation of SA in response to pathogen infection. The ICS pathway is known to be a major contributor to SA biosynthesis, particularly during pathogen-induced defense responses. Upon pathogen recognition, the activation of defense signaling pathways leads to an increase in ICS enzyme activity, resulting in the production of isochorismate and its subsequent conversion into SA. This pathogen-induced SA accumulation plays a crucial role in triggering and amplifying plant defense responses against pathogens [47].

The second route of SA biosynthesis, which is known as the PAL pathway, is responsible for the synthesis of only a small percentage of SA in plants such as *A. thaliana* and *Nicotiana benthamiana*, which were infected by pathogens or treated with UV during the reviewed studies [50]. This pathway occurs entirely within the cytosol. However, the PAL pathway has been known for much longer in relation to its role in the ICS pathway [48,51]. PAL is an upstream enzyme that leads to the production and accumulation of many other potentially defense-related compounds that are synthesized during the phenylpropanoid pathway [52]. The conversion of phenylalanine into *trans*-cinnamic acid is catalyzed by PAL, which has been identified in numerous plant species. The activity of this enzyme is tightly regulated by various biotic and abiotic stress factors [53]. Depending on the order of the hydroxylation of the aromatic ring in *trans*-cinnamate, SA can also be synthesized from *o*-coumaric acid or benzoic acid through chain-shortening reactions. The specific pathway that leads to SA formation is determined based on whether hydroxylation occurs before or after the chain-shortening reactions [50]. In certain plants, such as sunflower (*Helianthus annuus*), potato (*Solanum tuberosum*), and pea (*Pisum sativum*), SA is derived from benzoic acid. This result is achieved via the chain-shortening of cinnamate through a β-oxidation process, leading to the synthesis of benzoic acid, which is subsequently converted into SA [54]. On the other hand, *Primula acaulis* and *Gaultheria procumbens* can synthesize SA from *o*-coumarate [55].

## 5. Auxins

Auxins play a crucial role in the regulation of various aspects of plant growth and development. They are involved in controlling processes such as cell division, elongation, differentiation, tropisms (response to external stimuli), flowering, apical dominance (suppression of lateral bud growth), lateral root formation, senescence (aging), abscission (shedding of plant parts), and responses to environmental stresses [56]. The most well-studied auxin in plants is indole-3-acetic acid (IAA). The process of producing auxins in plants is highly intricate, and understanding this process can significantly enhance our comprehension of auxins’ biological role and contribution to the regulation of plant growth and physiology [57]. Although different plant species employ distinct strategies to optimize their metabolic pathways, it seems that there could be shared mechanisms for auxin biosynthesis, given that IAA is an phytohormone essential to plant life. Auxins are primarily generated in the plant’s apical meristems, young leaves, and flower buds, and they are then quickly transported throughout the plant via the phloem. Nonetheless, some research suggests that auxins may also be locally synthesized within the roots [58,59,60].

**Figure 5:**
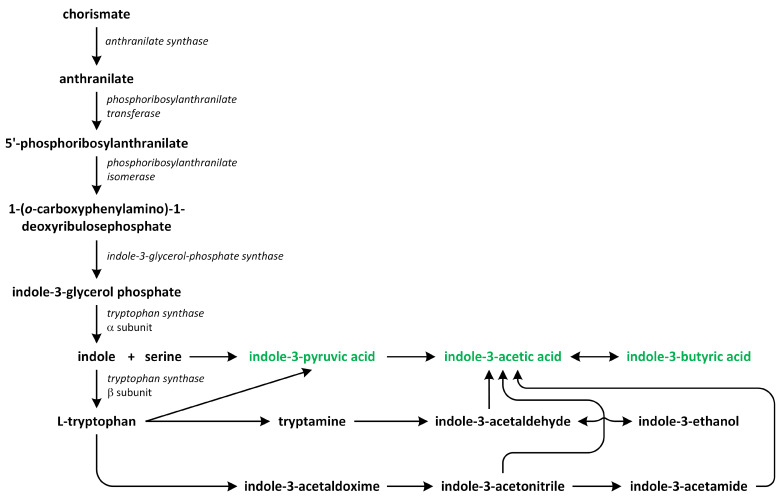
The biosynthetic pathways of auxins (phytohormones are green).

Two major routes have been assumed to contribute to de novo IAA biosynthesis in plants: the tryptophan (Trp)-dependent and Trp-independent pathways (Figure 5). Several pathways for Trp-dependent IAA biosynthesis have been proposed, including:

- the indole-3-acetamide (IAM) pathway;
- the indole-3-pyruvic acid (IPA) pathway;
- the tryptamine (TAM) pathway;
- the indole-3-acetaldoxime (IAOX) pathway [61,62,63,64,65].

Even though IAA was the first natural auxin to be identified, our understanding of the genes that encode all of the enzymes involved in auxin biosynthesis remains limited. It is also uncertain whether all of these pathways exist in all plant species [66].

Tryptophan, which is a precursor of the production of various indole-containing compounds in plants, including IAA, indole glucosinolates, phytoalexins, and tryptamine (TAM) derivatives, is synthesized from chorismate through the action of indole-3-glycerol phosphate synthase in the chloroplast. The *A. thaliana* genes *Anthranilate synthase1* (*AtASA1*) and *AtASA2* encode the α-subunit of the enzyme that is responsible for catalyzing the initial reaction through the Trp synthesis pathway. Indole-3-glycerol phosphate synthase, on the other hand, facilitates the conversion of 1-(*O*-carboxyphenylamino)-1-deoxyribulose-5-phosphate into indole-3-glycerol phosphate [67,68].

In hairy plant roots, IAA is synthesized from Trp via a reaction that consists of two steps. Tryptophan is first converted into IAM by the enzyme Trp-2-mono-oxygenase, and IAM is then transformed to IAA by indole-3-acetamide hydrolase [66]. This IAA biosynthetic pathway via IAM is a bacteria-specific synthesis because plant pathogens, such as *Pseudomonas* and *Agrobacterium*, have a large root-inducing plasmid and are able to induce hairy-root disease, which is characterized by a high root proliferation ratio from the place of infection, which occurs as a result of a high rate of IAA biosynthesis and accumulation [69]. *Pseudomonas* and *Agrobacterium* use Trp-2-mono-oxygeanse to convert Trp into IAM, which is then hydrolyzed into IAA. The Trp-dependent IAA biosynthesis pathway is the best-known and studied pathway used in this process [65]. It was reported that plants can also synthesize IAA via the IAM pathway. Therefore, enzymes amidases that are able to hydrolyze IAM into IAA have been identified in *A. thaliana*. This observation suggests that IAM may be a key substrate for IAA biosynthesis in plants [70]. In addition, overexpression of the *IAM* gene that encodes the enzyme tryptophan-2-mono-oxygeanse, which is involved in the conversion of Trp into IAM, increases the content of IAA and results in high-auxin phenotypes being present in plants, leading to increased hypocotyl elongation under light, severe rosette leaf epinasty, and apical dominance. Thus, plants are able to transform IAM into IAA, as has been shown in in vivo studies performed using *A. thaliana* [71]. IAM, which is an intermediate of auxin biosynthesis, can also be detected in many other plant species, regardless of whether they are monocots or dicots [66].

The indole-3-pyruvic acid (IPA) pathway occurs using intermediate IPA, which is crucial for auxin biosynthesis and plant development [60]. Tryptophan Aminotransferase of *Arabidopsis* 1 (TAA1) is a member of aminotransferases that can convert tryptophan into IPA. These enzymes have been identified in many plants. This observation indicates that this pathway is highly conserved. Mutations in the *TAA1* gene that encodes this protein lead to a high reduction in IAA levels. Thus, IPA-dependent IAA biosynthesis is an important route for the biosynthesis of free IAA. The TAA1 protein belongs to the superfamily of enzymes that is dependent on the α class of PLP and exhibits Trp aminotransferase activity, which uses L-Trp, but not D-Trp, as a substrate. This enzyme can also react with other amino acids, such as L-phenylalanine, tyrosine, leucine, alanine, methionine, and glutamine [72].

Tryptophan can also be converted into TAM through the action of cytosolic Trp decarboxylase (TDC). TAM is a protoalkaloid that is involved in an early step of the biosynthetic pathway that influence terpenoid indole alkaloids [66]. TDC has been functionally and structurally characterized. It participates in synthesis of indole alkaloids and serotonin in many plants. However, TDC members probably do not fully contribute to IAA biosynthesis. As observations show, overexpression of the *TDC* gene in *Catharanthus roseus* has been indicated to generate increased levels of TAM, but not IAA, accumulation in tobacco (*Nicotiana tabacum*) [73]. Moreover, genetically modified rice plants with overexpression of the *TDC* gene displayed a typical phenotype and contained serotonin levels that were 25 times higher in leaves and 11 times higher in seeds than those found in their wild-type equivalents [74]. In rice, TDC can be involved in the production of metabolites that are derivatives of the TAM-inducing disease known as Sekiguchi lesions or the accumulation of serotonin, which is a well-known neurotransmitter in mammals and a signalling molecule that is present in many plants [75].

Another possible pathway involved in the synthesis of IAA from Trp consists of the conversion of indole-3-acetaldoxime into indole-3-acetonitrile (IAN). In this reaction, amino nitrogen is removed from tryptophan via hydrolysis of the nitrile to form acid [76]. Indole-3-acetaldoxime (IAOX) is a precursor of the biosynthesis pathway of IAA and its conjugates, and it has been proposed as the branch point between the biosynthesis of IAA and indole-3-methyl glucosinolate [77].

Studies of *A. thaliana* mutant lines show that IAOX is a key intermediate involved in IAA biosynthesis. This compound can be converted into IAN by eliminating one molecule of water. IAN is subsequently transformed into IAA by a family of nitrilases. IAOX can be hydrolyzed to form indole-3-acetaldehyde, and the aldehyde can then be converted into IAA via an action of two enzymes: aldehyde oxidase (AO1) or aldehyde dehydrogenase. AO1 is important enzyme for auxin biosynthesis [78]. The enzyme AO1 shows substrate specificity to indole-3-acetaldehyde, and the regulation of the activity of this enzyme is unaffected by the high levels of IAA. However, plant mutants that are unable to perform molybdopterin biosynthesis, which is an essential cofactor of all aldehyde oxidases, did not show phenotypes that were characteristic of auxin deficiency. This result suggests that aldehyde oxidases are probably not essential for auxin biosynthesis, as was previously believed [79].

## 6. Melatonin

Melatonin, which is formally known as *N*-acetyl-5-methoxytriptamine, is an indolamine plant hormone that plays a key role in controlling growth and various physiological responses. Affected functions include root structure and development, flowering, leaf aging, fruit maturation, and determining the levels of chlorophyll, proline, and carbohydrates. It also functions as a signaling molecule during non-biological stress situations, like cold, drought, heavy metal exposure, UV radiation, and salinity, and it helps to direct plant defense responses against pathogen invasions [80,81]. The highest levels of melatonin in plants are found in the mitochondria, endoplasmic reticulum, and chloroplasts, suggesting that these organelles are the primary sites involved in its biosynthesis. The varied locations of melatonin within the plant could potentially influence its mode of action in different ways [82].

**Figure 6:**
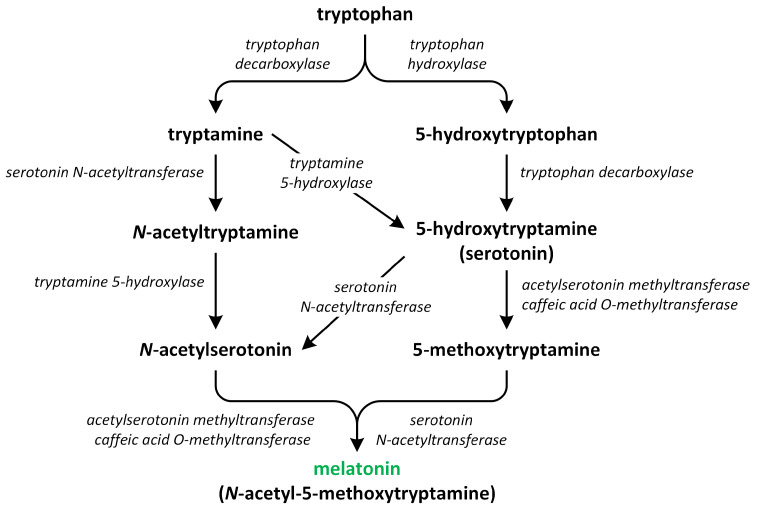
The biosynthetic pathways of melatonin (phytohormone is green).

The biosynthesis of melatonin begins with an essential aromatic amino acid tryptophan, which is synthesized de novo via the shikimate pathway (Figure 6).

The pathway involved in the synthesis of this phytohormone occurs through various four-step reactions catalyzed by six enzymes: tryptophan decarboxylase (TDC), tryptophan hydroxylase (TPH), tryptamine 5-hydroxylase (T5H), serotonin *N*-acetyltransferase (SNAT), *N*-acetylserotonin *O*-methyltransferase (ASMT), and caffeic acid *O*-methyltransferase (COMT) [83]. These enzymes have different cellular localization. Chloroplasts and mitochondria contain SNAT. TPH, ASMT/COMT, and TDC are present in the cytoplasm. T5H can be only found in the endoplasmic reticulum [84]. Recent research suggests that the synthesis of melatonin in plants primarily takes place in chloroplasts and mitochondria. These organelles have inherited the ability to produce melatonin from their bacterial forebears. Under normal circumstances, melatonin production predominantly occurs in the chloroplasts. However, if the pathway in the chloroplasts is obstructed, the production of melatonin shifts to the mitochondria. Therefore, under stress conditions, melatonin is chiefly synthesized in the mitochondria [85,86].

The first step of melatonin biosynthesis is the formation of serotonin from Trp. Two different pathways involved in Trp and serotonin synthesis were identified in vascular plants. The first pathway involves the decarboxylation of tryptophan into tryptamine via the reaction catalyzed by the enzyme TDC. Next, tryptamine is hydroxylated into serotonin via the action of an enzyme called T5H. Another possibility is that the hydroxylation of Trp into 5-hydroxytryptophan via the action of enzyme TPH occurs. Next, the decarboxylation of 5-hydrotryptophan into serotonin by TDC takes place. The enzyme TDC is localized in the cytoplasm and characterized by a good affinity for two substrates: tryptophan and 5-hydroxytriptophan; thus, both pathways can be present in plants at levels of equal importance. The activity of TDC is considered to be the rate-limiting step in melatonin biosynthesis [80,87,88,89].

Thereafter, serotonin is transformed into melatonin via a two-step reaction. The roles of three different enzymes—SNAT, ASMT, and COMT—in this reaction were confirmed. These enzymes may possess various biochemical isoforms. SNAT is responsible for N-acetylation of serotonin, which is further methylated by the methyltransferases ASMT or/and COMT, leading to production of melatonin [90]. In addition, SNAT can convert serotonin directly into *N*-acetylserotonin through alternative minor pathways. The enzymes ASMT and COMT catalyze the conversion of serotonin into 5-methoxytryptamine. This compound is then changed into melatonin by the enzyme SNAT. These three enzymes are characterized by an affinity for substrates such as serotonin, *N*-acetylserotonin, and 5-methoxytryptamine; therefore, the order in which the different enzymes act differs [81]. SNAT is localized in the chloroplasts, whereas ASMT and COMT are present in the cytoplasm [91]. The biosynthetic ratio connected to the transformation of tryptophan into serotonin is considerably higher in relation to the conversion of serotonin into melatonin, leading to a low level of this phytohormone being present in plants [83].

When plants grow under normal environmental conditions and/or serotonin is pre-sent in low levels in cells, two following pathways exist in plants: (i) tryptophan → trypta-mine → serotonin → *N*-acetylserotonin → melatonin; and (ii) tryptophan → 5-hydroxytryptophan → serotonin → *N*-acetylserotonin → melatonin. However, the following reactions, which lead to the production of melatonin from tryptophan, were detected in plants that produce large amounts of serotonin during the senescence process. Furthermore, experiments performed on rice seedlings showed that exogenously applied *N*-acetylserotonin can be converted into serotonin by the enzyme *N*-acetylserotonin deacetylase, resulting in a reduction in the content of melatonin in plants. These different pathways and sites of melatonin synthesis suggest that the production of melatonin is highly diverse in plants [80,85,86,88].

## 7. Abscisic Acid

Abscisic acid (ABA) also plays an important role in many cellular processes, including seed development, dormancy, germination, vegetative growth, and responses to environmental stresses [92]. ABA is ubiquitous in lower and higher plants. It was reported that young tissues contain high levels of ABA [93]. The biosynthesis of ABA predominantly takes place in vascular tissues, and it is subsequently transported into target tissues. This transportation process occurs through both the xylem and phloem, enabling bidirectional transport between the roots and shoots. The naturally occurring form of ABA is *S*-(+)-ABA, which has a side chain defined as 2-*cis*, 4-*trans*. The *trans*-ABA is biologically inactive, whereas *R*-(−)-ABA, which can potentially result from racemization through the catabolite ABA-*trans*-diol, exhibits biological activity [94,95].

The initial stages of ABA biosynthesis occur within plastids. In plants, two distinct ABA biosynthesis pathways have been identified. The first pathway is a direct route, while the second pathway is indirect and involves the conversion of a C_15_ compound (i.e., farnesyl pyrophosphate) and a C_40_ carotenoid [96]. Through the characterization of ABA-deficient mutants and the isolation of relevant mutated genes, it has been determined that the indirect pathway is the primary route involved in ABA biosynthesis in plants [96,97]. Investigations into the biosynthesis of carotenoids, as well as their pathways and mechanisms, have indicated that mevalonate (MVA) plays a minor role as a precursor of carotenoid biosynthesis in chloroplasts [98,99].

**Figure 7:**
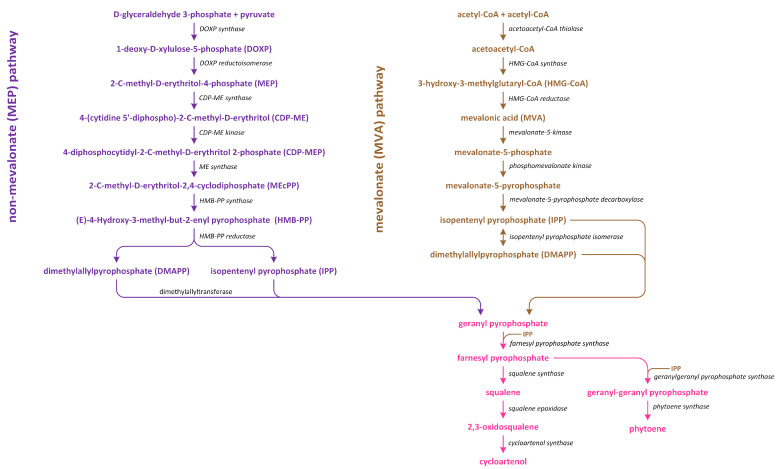
The methylerythritol phosphate (MEP) and mevalonate (MVA) pathways lead to the conversion of isopentenyl diphosphate to cycloartenol and phytoene.

In plants, there are two distinct pathways involved in isoprenoid production (Figure 7). The first pathway, which is known as the MVA pathway, is shared by animals, fungi, and a few bacteria. It operates in both the cytosol and mitochondria, and it is responsible for generating precursor molecules used in the synthesis of sterols, certain sesquiterpenes, and the side chain of ubiquinone. On the other hand, the second pathway, which is called the methylerythritol phosphate (MEP) pathway, is localized within plastids [100].

The MVA pathway initiates in tandem with the condensation of three acetyl~CoA molecules (Figure 7). Subsequently, a reduction process leads to the formation of MVA, which undergoes two phosphorylation reactions to produce mevalonate-5-diphosphate. Further phosphorylation, which is followed by decarboxylation-driven dephosphorylation, yields isopentenyl pyrophosphate (IPP). This compound can be isomerized to create dimethylallyl pyrophosphate (DMAPP), which serves as a precursor of the biosynthesis of various isoprenoid-derived phytohormones [101]. On the other hand, the MEP pathway initiates in tandem with the condensation of pyruvate and glyceraldehyde 3-phosphate (Figure 7). This condensation reaction gives rise to 1-deoxy-D-xylulose-5-phosphate (DOXP). Subsequently, DOXP is converted into methylerythritol phosphate. Methylerythritol phosphate then undergoes conjugation with the cytidyl phosphate moiety, followed by another phosphorylation step, i.e., the release of CMP, and cyclization. The resulting product is subjected to a reduction process, leading to the formation of (*E*)-4-hydroxy-3-methyl-but-2-enyl diphosphate (HMBDP). HMBDP can be further reduced to produce IPP and, to a lesser extent, DMAPP. Both HMBDP and DMAPP can serve as precursors of cytokinin synthesis, though it is worth noting that the level of HMBDP in plastids is approximately five times higher than that of DMAPP [102]. MVA can be converted into an intermediate IPP (in cytosol), which is an intermediate in both carotenoid and steroid pathways. Furthermore, in plastids in which carotenoid synthesis occurs, IPP is generated through the MEP pathway using pyruvate and glyceraldehyde-3-phosphate. The enzyme responsible for catalyzing the initial step in the non-MEP biosynthetic pathway is called DOXP synthase (DXS). After IPP is formed, it is converted into a C_20_ compound known as geranylgeranyl pyrophosphate (GGPP) [103].

**Figure 8:**
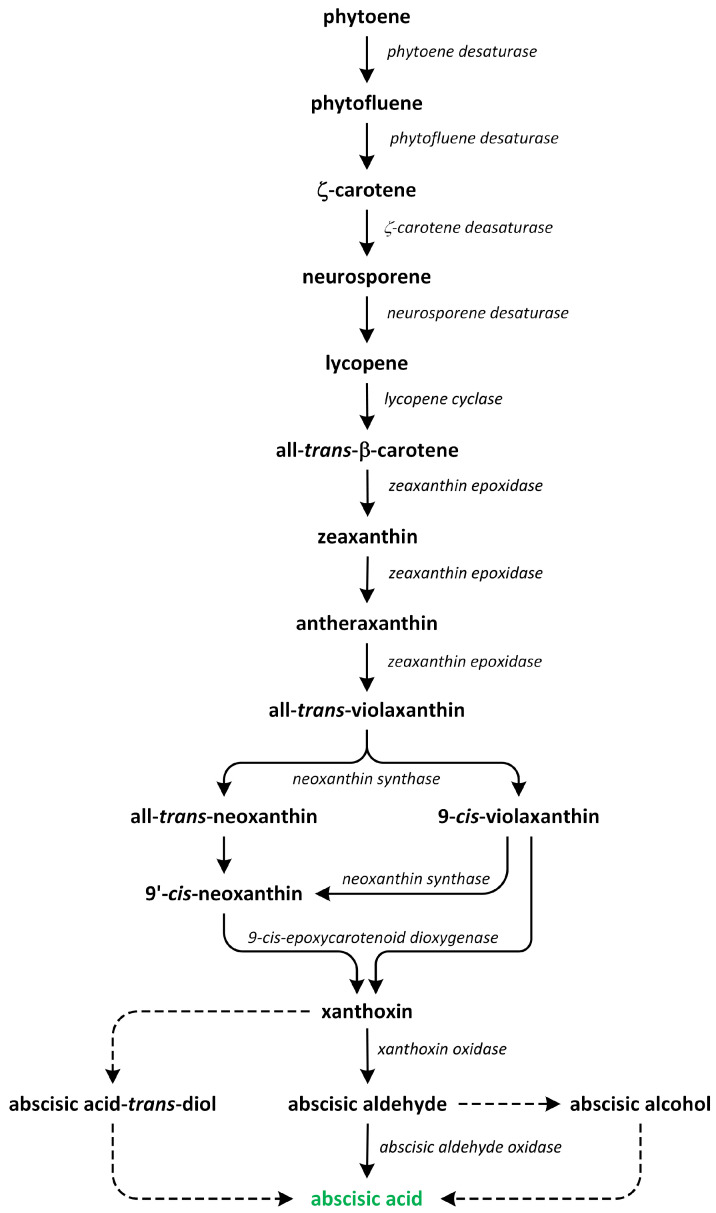
The biosynthetic pathways of abscisic acid (phytohormone is green).

The conversion of geranylgeranyl pyrophosphate (GGPP) into phytoene, which is a C40 carotenoid, is the initial and rate-limiting step involved in carotenoid synthesis. This reaction is catalyzed by the enzyme phytoene synthase (Figure 8).

Subsequently, phytoene undergoes a series of enzymatic conversions in a sequential order, resulting in the formation of ζ-carotene, lycopene, β-carotene, and zeaxanthin [104]. The subsequent step, which is specific to the ABA biosynthesis pathway and occurs within plastids, involves the epoxidation of zeaxanthin and antheraxanthin to produce violaxanthin. This enzymatic conversion is mediated by zeaxanthin epoxidase. Following this step, the oxidative cleavage of xanthophylls, such as 9-*cis*-violaxanthin and/or 9’-*cis*-neoxanthin, occurs, leading to the formation of xanthoxin. This step is catalyzed by 9-*cis*-epoxycarotenoid dioxygenase and subsequently exported to the cytosol. Once 9-*cis*-epoxycarotenoids are cleaved, xanthoxin is further converted into ABA [96].

Three possible pathways have been proposed for the final stages of ABA biosynthesis that involves abscisic aldehyde, xanthoxic acid, or abscisic alcohol acting as intermediates. In the first proposed pathway, the enzyme encoded by the *AtABA2* gene, which is a short-chain alcohol dehydrogenase/reductase, catalyzes the initial step and produces ABA aldehyde. The final step in this biosynthetic pathway is catalyzed by ABA aldehyde oxidase. The activities of aldehyde oxidase and xanthine dehydrogenase require molybdenum cofactor sulfurase, which is responsible for the sulfurylation of a dioxo form into a sulfurylated mono-oxo form, with L-cysteine used as a sulfur donor [94,105].

The second pathway of ABA biosynthesis that involves xanthoxic acid was discovered in ripening avocado (*Persea americana*) fruits. In these experiments, inhibition of aldehyde oxidase activity by tungstate, which is a potent inhibitor of molybdoenzymes in plants, led to the accumulation of xanthoxin. This result indicates that xanthoxin is a substrate for aldehyde oxidase. It is possible to speculate that the two ABA biosynthetic pathways may operate in an organ- and/or developmental stage-dependent manner, suggesting a potential regulatory mechanism [96,98,106].

The last ABA biosynthetic pathway, which involves abscisic alcohol, can be activated in certain mutants. It was shown that in the *flacca* and *sitiens* mutants of tomato (*Solanum lycopersicum*), when abscisic aldehyde was supplied externally, it was converted into abscisic alcohol. Subsequently, abscisic alcohol was oxidized to ABA (Figure 8) [107]. This reaction is a non-specific source of ABA in wild-type plants, though it may play a more significant role in mutants that have impaired capacity to directly convert abscisic aldehyde into ABA. This finding suggests that the shunt pathway serves as an alternative route for ABA production in tomato mutants [96].

## 8. Brassinosteroids

Brassinosteroids (BRs) are steroid phytohormones that elicit a wide spectrum of morphological and physiological responses, as well as a tolerance of abiotic and biotic stress [108,109,110,111,112,113,114]. BRs are categorized into three primary groups based on the number of carbon atoms present in each steroid molecule, and BRs are divided into three main groups on the basis of each steroid molecule, i.e., C_27_, C_28_, and C_29_ [115,116]. The basic structure of C_27_-BRs is the 5α-cholestane skeleton; 5α-ergostane serves as the foundational structure of C_28_-BRs, while 5α-stigmastane forms the basis of C_29_-BRs. The structures of these hormones vary due to the type and orientation of oxygenated functions in the A- and B-rings, as well as the number and position of functional groups in the side chain. These variations arise from oxidation and reduction reactions during their synthesis in plants [117,118,119].

**Figure 9:**
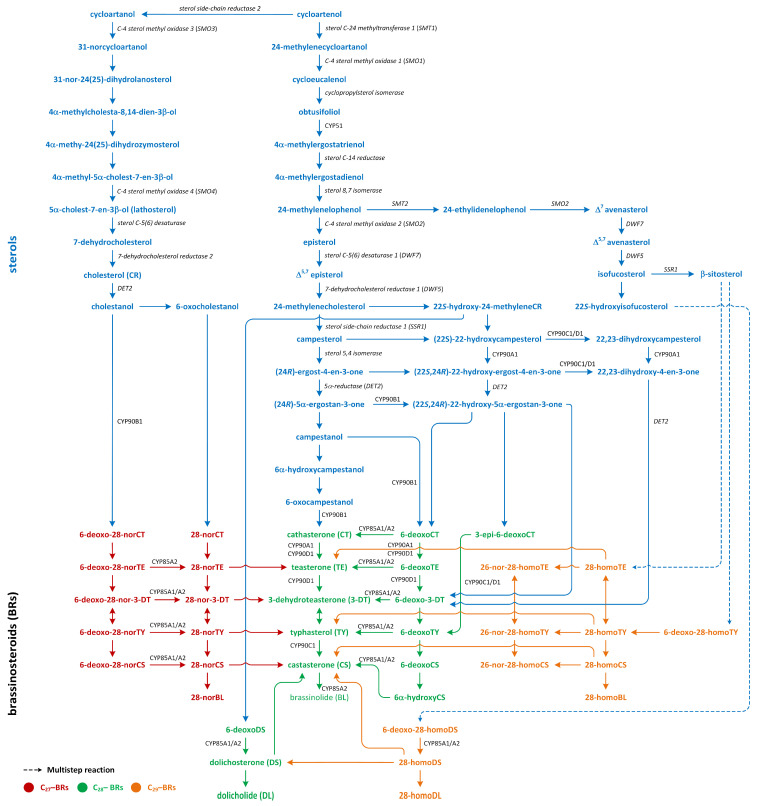
The biosynthetic pathways of different types of brassinosteroids (phytohormones are red, green, and orange).

Three pathways of BR biosynthesis that lead to the production of BRs of type C_27_, C_28_, or C_29_ were identified in plants. Early steps involved in their synthesis, which are common for all BRs, occur via the MVA or non-MVA (MEP) pathways (Figure 7). Later steps differentiate between the biosynthesis pathways (cycloartenol- and cycloartanol-dependent) of this group of plant hormones (Figure 9) [116,120,121].

Isopentenyl pyrophosphate (IPP), which is an intermediary compound involved in the steroid synthesis pathway (for example, cholesterol), can be created via two routes: the MEP pathway (in lower plants) and the MVA pathway (in most higher plants) (Figure 7) [122]. The conversion of IPP into geranyl pyrophosphate and, subsequently, farnesyl pyrophosphate leads to the creation of squalene (Figure 7). The squalene is then oxidized to create squalene-2,3-oxide under the influence of the enzyme squalene epoxidase. Following this step, cycloartenol synthase transforms squalene-2,3-oxide into cycloartenol, which is a crucial substrate used in the synthesis of C_27_, C_28_, and C_29_-BRs. Cycloartenol can then be transformed into cycloartanol and, through a series of reactions, into cholesterol/cholestanol and/or 6-oxocholestanol, leading to the creation of C_27_-BR (Figure 9). In another process, cycloartenol may serve as a substrate for the C-24 methylation reaction, which is catalyzed by sterol C-24 methyltransferase (SMT1), resulting in the production of 24-methylenecycloartanol. Subsequent reactions, which are driven by enzymes such as C-4 sterol methyl oxidase (SMO1), cyclopropylsterol isomerase, obtusifoliol 14α-demethylase (CYP51), and sterol C-14 reductase, lead to the formation of 4α-methylergostatrienol. The intermediates of these reactions include cycloeucalenol and obtusifoliol. Next, the enzyme sterol C-14 reductase catalyzes the reduction in 4α-methylergostatrienol to create 4α-methylergostadienol, which is subsequently transformed into 24-methylenelophenol by the enzyme sterol Δ^7^-isomerase. 24-methylenelophenol can serve as a substrate involved in two distinct sterol biosynthesis pathways. The first pathway leads to the synthesis of isofucosterol/β-sitosterol, which are precursors of C_29_-BRs. 24-methylenelophenol is converted by sterol methyltransferase 2 (SMT2) into 24-ethylidenelophenol. This compound is then converted into avenasterol by sterol methyl oxidase 2. Next, avenasterol is transformed into isofucosterol and β-sitosterol, which is hydroxylated into 6-deoxo-28-homoTY and then oxygenated into 28-homoTY by CYP724B2 and CYP90B3 C-22 hydroxylase, respectively [123]. 28-homoTY can also be generated from 28-homoTE, though the intermediates involved in this reaction have not yet been identified [116]. 28-homoTY is transformed into 28-homoCS and 28-homoBL via the actions of enzymes CYP85A1/A2 oxidases. Furthermore, 28-homoDL and CS can be produced from isofucosterol using a series of intermediates: 22-hydroxyisofucosterol, 6-deoxo-28-homoDS, and 28-homoDS. Castasterone can also be synthesized from β-sitosterol using intermediary compounds such as 22-homositosterol, 6-deoxohomositosterol, and 28-homoCS. It has been discovered that 28-homoTE, 28-homoTY, and 28-homoCS can be converted into 26-nor-28-homoTE, 26-nor-28-homoTY, and 26-nor-28-homoCS, respectively [116,124,125,126,127,128].

In the second pathway, 24-methylenelophenol is converted into campesterol, which acts as a precursor for C_28_-BRs (Figure 9) [122,129,130]. In this C_28_-BRs biosynthesis, two parallel pathways, i.e., the late and early C-22 oxidation pathways, occur. Campesterol is then converted into (24*R*)-ergostan-4-*en*-3*β*-one and campestanol (CN). In the parallel early C-22 oxidation pathway, C-22α hydroxylation of 24-methyleneCR by the enzyme C-22*α* hydroxylase leads to the biosynthesis of 22-hydroxy-24-methyleneCR [131]. Subsequent processes resemble the late C-22 oxidation pathway, resulting in the generation of 22-hydroxy variants of the related substances. The distinguishing aspect between the C-22 oxidation sub-pathways is the formation of 6-deoxocathasterone (6-deoxoCT) from (22S,24R)-22-hydroxy-5α-ergostan-3-one, circumventing the synthesis of CN. This process is referred to as the CN-independent route of BR biosynthesis. Nonetheless, during each phase of the late C-22 oxidation pathway, the compound involved could undergo hydroxylation via C-22α hydroxylase, leading to the formation of hydroxylated versions of compounds that are part of the early C-22 pathway [132]. Transformation in 22-hydroxymethyleneCR results in the creation of 6-deoxodolichosterone, which is converted into dolichosterone (DS), dolicholide (DL), castasterone (CS), and brassinolide (BL) [120,128].

The biosynthesis of C_27_-BR involves the late C6 oxidation pathway. In these reactions, cholesterol is converted into cholestanol by the 5α-reductase enzyme DET2. Consecutive reactions then produce a sequence of compounds: 6-deoxo-28-norcathasterone, 6-deoxo-28-norteasterone, 6-deoxo-28-nor-3-dehydroteasterone, 6-deoxo-28-nortyphasterol, and 6-deoxo-28-norcastasterone. In addition, the early C6 oxidation pathway starts with the oxidation of cholestanol to create 6-oxocholestanol. This process leads to the synthesis of a series of compounds, including 28-norcathasterone, 28-norteasterone, 28-nor-3-dehydroteasterone, 28-nortyphasterol, 28-norcastasterone, and, eventually, 28-norbrassinolide [124,133,134].

The late C6 pathway begins with the hydroxylation of CN to form 6-deoxocathasterone (6-deoxoCT), which is a process catalyzed by the enzyme 22α-hydroxylase. Interestingly, 6-deoxoCT can also be directly synthesized from 22-hydroxy-5α-ergostan-3-one. Following this step, 6-deoxoCT can be hydroxylated by C-23 hydroxylase to form 6-deoxoteasterone. This compound then undergoes C-3 oxidation to form 3-dehydro-6-deoxoteasterone (6-deoxo-3-DT) under the action of CYP90D C3-oxidase. Next, 6-deoxo-3-DT is converted into 6-deoxotyphasterol (6-deoxoTY) by the D11 CYP724B1 enzyme. In the subsequent step, 6-deoxoTY is hydroxylated to form 6-deoxocastasterone (6-deoxoCS) by the enzyme 2α-hydroxylase. 6-deoxoCS is then converted into castasterone (CS) by the enzymes BR-6-oxidase1 and BR-6-oxidase2. Afterward, CS is transformed into brassinolide (BL) via a process called Baeyer–Villiger oxidation, which is facilitated by the enzyme BR-6-oxidase2 (CYP85A2). At the start of the early oxidation pathway, CN is hydrated to form 6α-hydroxyCN. This compound is then oxidized to create 6-oxo-CN. The enzyme 22α-hydroxylase is responsible for the conversion of 6-oxo-CN into cathasterone (CT). CT then undergoes a series of transformations to form teasterone (TE), 3-dehydroteasterone, typhasterol (TY), CS, and, finally, BL [112,116,120,124,125,132,135].

## 9. Cytokinins

Cytokinins (CKs) regulate plant development, nutrient uptake, cell division, cell differentiation, chlorophyll senescence, apical dominance, embryonic development, and general aspects of plant growth [136]. Cytokinins are *N*^6^-substituted adenine derivative compounds that can be divided into two groups: (i) isoprenoid and (ii) aromatic CKs. Isoprenoid CKs more abundant than aromatic compounds [137,138]. Isoprenoid CKs include compounds such as *N*^6^-(∆^2^-isopentenyl adenine (iP), dihydrozeatin, and *trans*- and *cis*-zeatin (*t*Z and *c*Z, respectively), while *N*^6^-benzyladenine and its hydroxyl derivatives, such as *ortho*- and *meta*-topolin, are representatives of aromatic CKs [139].

Cytokinin levels are spatially and temporally regulated. Cytokinins are abundant in the apical meristems, root tips, and immature seeds. It is generally assumed that the root tip is the main site of the biosynthesis of CKs. On the other hand, literature data indicate that the shoot apex, cambium, and immature seeds are also capable of synthesizing CKs [140]. Changes in CK levels are related to the phase of cell cycle, consequences of influence of environmental factors, presence or lack of mineral nutrients, and effect of abiotic or biotic stress factors. The levels of active CKs in plants are highly regulated by the rates of production, interconversion, transport, and degradation [141].

Cytokinins can be synthesized de novo as nucleotide mono-, di-, or tri-phosphates, which are characterized by low biological activity. Moreover, tRNA is a source of CKs. The release of cytokinins from this nucleic acid leads to the creation of nucleotide monophosphates. The conversions of nucleotides, nucleosides, and free bases are catalyzed by enzymes involved in the metabolism of adenine [142]. In the next step, an isoprenoid moiety is added to the adenine present in the ATP and/or ADP molecule [143]. An alternative pathway, in which a hydroxylated side chain is added to the adenine moiety, has also been proposed [144].

DMAPP and HMBDP are the common isoprenoid side chain donors in biosynthetic pathway of CKs [102,145]. In the case of formation of isopentenyladenine-type CKs via the precipitation of DMAPP, the side chain is hydroxylated by cytochrome P450 mono-oxygenase (Figure 7) [146]. The CK nucleotides that include nucleotides released from tRNA are then hydrolyzed to free bases [102].

**Figure 10:**
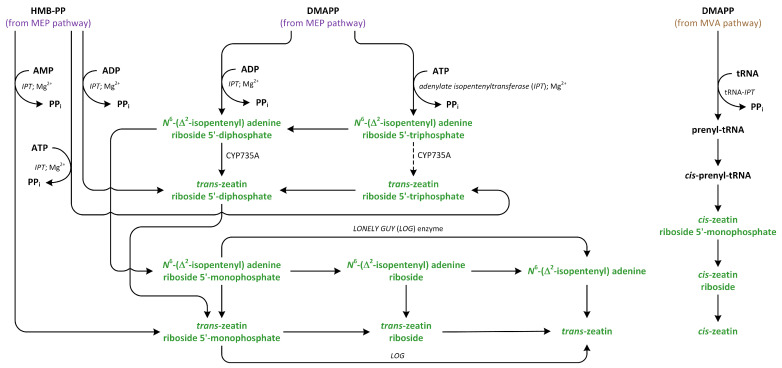
The biosynthetic pathways of cytokinins (phytohormones are green).

Cytokinin biosynthesis is catalyzed by the enzyme isopentenyl transferase (IPT) (Figure 10). There are two types of the adenylate IPT, which are responsible for attachment of an isopentenyl group to the *N*^6^ atom present in AMP, ADP, or ATP and tRNA. IPT does not add isopentenyl group to adenosine or adenine. IPT acts in the same way on adenine present in the structure of tRNA [143]. Adenylate IPT can synthesize iP and *t*Z nucleotides [145,147].

Isopentenyl transferase isolated from vascular plants preferentially acts on ADP or ATP, rather than AMP, as prenyl acceptors and almost exclusively uses DMAPP as a prenyl donor, which, in turn, can synthesize iP riboside 5′-diphosphate or iP riboside 5′-triphosphate. Past kinetic studies of the activity of IPT in *A. thaliana* showed that the Km values for AMP are much higher than those of ADP and ATP, which were used as substrates. Moreover, the cellular level of AMP is low, indicating that AMP is not an essential substrate involved in IPT activity [143]. Interestingly, IPT isolated from mulberry (*Morus alba*) can also recognize and react with dATP, dADP, CDP, and GDP as substrates. In the case of GDP, the isopentenyl moiety is added to *N*^2^ group (the exocyclic nitrogen), rather than *N*^1^ group (the endocyclic nitrogen), which is closer to *C*^6^ [148,149]. However, IPT that is isolated from *Agrobacterium* cells, which is called Tmr, uses AMP as an acceptor and HMBDP or DMAPP as donors in vitro, while HMBDP is predominantly utilized in vivo, leading to the production of *t*Z, which is a highly active CK, in plastids of host plants [150]. IPTs are transported into different organelles, such as mitochondria, plastids, and the cytosol. Therefore, isoforms of IPT, such as AtIPT1, AtIPT3, AtIPT5, and AtIPT8, are present in the plastids and involved in the production of CKs from DMAPP that originate from the MEP pathway [151]. On the other hand, AtIPT4 is localized in cytosol, whereas AtIPT7 can be found in mitochondria. These observations suggest that DMAPP involved in CK biosynthesis is also derived via the MVA pathway [147].

Studies suggest that tRNA isopentenyltransferase (tRNA IPT) is responsible for the transportation of DMAPP via MVA pathway to adenine in tRNA, which results in the creation of *c*Z [143]. Studies of the synthesis of the isopentenyl side chain of the CK present in the tRNA molecule in vitro demonstrated that the isopentenyl group was derived from CKs MVA, and the turnover of CK-containing tRNA may serve as a minor source of free *c*Z in plant cells. It has been proposed that the target adenine is released from the tRNA structure in a similar way to that modified by other nucleic acid-editing enzymes. This type of IPT has been identified in almost all living organisms, including bacteria, yeast, animals, and plants. However, tRNA IPT was not found in Archaea. The isopentenylation of adenine present in tRNA has an effect on translational efficiency and precision. It improves proofreading during the translation process by decreasing misreading at the first position of the codon [146,152]. The different origins of large amounts of DMAPP utilized in biosynthesis of CKs, such as *t*Z and *c*Z, suggest that plants can precisely regulate the homeostasis of these CKs [147].

## 10. Gibberellins

Gibberellins (GAs) are tetracyclic and diterpenoid plant hormones that form a large group of carboxylic acids [153]. GAs are present in all vascular plants and lower plants, such as lycophytes and ferns. GAs are involved in the promotion of organ growth, enhance cell elongation and/or division, and activate most developmental processes (i.e., seed germination, induction of flowering, and maturation) in many plant species [154,155]. GAs were first isolated from the fungus *Gibberella fujikuroi* (reclassified as *Fusarium fujikuroi*), which stimulated growth in infected vascular plants. Next, their presence in plants as phytohormones was confirmed in the late 1950s. The system that numbers GAs, starting with GA_1_, assigns GAs in order of their discovery and structural characterization [156]. The most well-known and biologically active GAs in plants are GA_1_, GA_3_, GA_4_, and GA_7_. There are three structural properties that alter these GAs: the presence of hydroxyl group in C-3β, carboxyl group in C-6, and lactone between C-4 and C-10. The 3β-hydroxyl group can be replaced by other functional groups at positions C-2 and/or C-3. However, GA_5_ and GA_6_ are representatives of bioactive GAs that lack a hydroxyl group at C-3β [153].

The knowledge of GA biosynthesis has developed rapidly in recent years. Quantita-tive analysis shows that biosynthesis of GAs occurs in actively growing tissues, i.e., young leaves, shoot apices, and flowers. In contrast, there are some reports that indicate that xylem and phloem exudates contain Gas, suggesting the presence of long-distance transportation of these phytohormones in plants [154,157,158].

In plants, GAs are formed from GGPP through IPP, which represents the C-5 building moiety involved in the synthesis of terpenoid (isoprenoid) compounds (Figure 7) [155,159]. In the green tissues of plants, IPP is produced via two pathways: (i) the MVA pathway in the cytoplasm and (ii) the methylerythritol phosphate (MEP) pathway in plastids in two-step reactions. Such a metabolism is ancient and leads to the synthesis of 12,000 natural diterpenoid products in plant tissues. Recent data suggest that this compound is cyclized to the tetracyclic hydrocarbon precursor *ent*-kaurene in plastids by *ent*-copalyl diphosphate, which predominantly occurs through the MEP pathway [157]. However, the MVA pathway may also be involved in the synthesis of isoprenoid intermediates of GGPP and their transport from the cytosol into the plastids [160]. The formation of *ent*-kaurene from GGPP takes place in the stroma of proplastids or in the developing chloroplasts, except those of mature chloroplasts [161]. Ten functional GGPP synthase (GGPPS) genes were discovered in *A. thaliana*. Among these genes, seven encode enzymes were localized in the plastid. However, GGPPS11 is expressed most strongly and constitutively in plants that produce most of the substrate utilized in the biosynthesis of various terpenoids in plastids [162]. In contrast to *A. thaliana*, rice is reported to contain one functional GGPPS that is present in the plastid, which is involved in the biosynthesis of all diterpenoid compounds, including *ent*-kaurene, which is a key intermediate involved in the formation of GAs [163].

**Figure 11:**
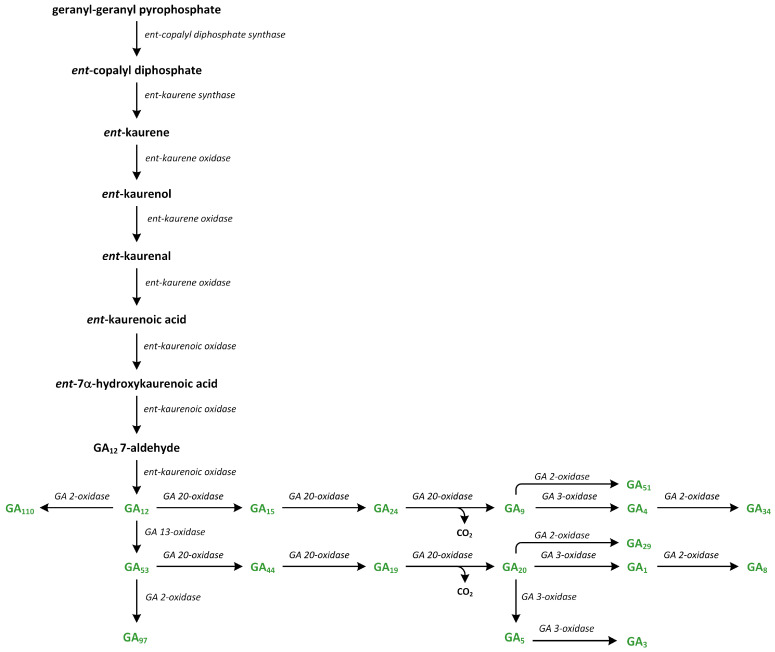
The biosynthetic pathways of gibberellins (phytohormones are green).

Firstly, the synthesis of *ent*-kaurene from GGPP starts via cyclization, which initiated by the proton of the dicyclic *ent*-copalyl diphosphate (CPP), which is catalyzed by a type II diterpene cyclase: *ent*-copalyl diphosphate synthase (CPS) (Figure 11). CPS contains a conserved DXDD motif, in which aspartate gives a proton to initiate this reaction via cyclization. Additionally, a water molecule attached to amino acids (histidine and asparagine) plays role as the catalytic base that accepts a proton and terminates the reaction [164]. CPS activity is inhibited by high concentrations of both Mg^2+^ and GGPP, which are promoted by light, leading to reduced flux into the GA pathway during de-etiolation. This process is part of the mechanism used to decrease GA biosynthesis and its cellular concentration [165].

The second step of the transformation of *ent*-copalyl diphosphate to *ent*-kaurene is catalyzed by a type I cyclase, which is known as *ent*-kaurene synthase (KS) (Figure 11). Cyclization reaction is initiated via metal-dependent heterolytic cleavage of the O-C bond. A pimeren-8-yl carbocation takes place, which then undergoes rearrangement. The loss of H^+^ is important step that leads to tetracyclic *ent*-kaurene [166]. Enzyme KS contains RLX(N,D)DXX(S,T,G)XXX(E,D) and DDXXD motifs, which can bind to ion Mg^2+^, which is associated with the diphosphate residue and participates in its ionization [167]. The lycophyte *Selaginella moellendorfii*, which is one of the first plants to have evolved the ability to synthesize GAs, possesses monofunctional CPS and KS enzymes, which are characteristic of vascular plants. In *S. moellendorffii*, KS is characterized by low substrate specificity, as it converts different stereoisomeric forms of CPP to various organic compounds, while KS enzymes in angiosperms are specific only for *ent*-CPP [168]. In angiosperms, CPS and KS possess the functional diversification required to produce the diterpenoids involved in plants’ defense responses [157,169].

GA_12_, which is the first C_20_-GA, is formed from *ent*-kaurene, and the reactions of these synthetic steps of GA productions are catalyzed by the following enzymes: *ent*-kaurene oxidase (KO), two cytochrome P450 mono-oxygenases, and *ent*-kaurenoic acid oxidase (KAO) (Figure 11) [170]. In *A. thaliana*, KO is associated with the outer chloroplast membrane and the endoplasmic reticulum. On the other hand, two KAOs are located in the endoplasmic reticulum. This observation suggests that *ent*-kaurene is oxidized and then transported from plastids to the endoplasmic reticulum [157]. KO catalyzes the oxidation of *ent*-kaurene into *ent*-kaurenoic acid via repeated hydroxylation of C-19. The intermediate aldehyde, i.e., *ent*-kaurenal, is present in this reaction [171]. The first reaction of hydroxylation to *ent*-kaurenol is the rate-limiting step in the GA biosynthesis pathway [172]. The reactions catalyzed by KAO have been studied in cells that were isolated from developing seeds, i.e., endosperm [158].

In plants, *ent*-kaurenoic acid is converted into GA12 in three steps that involve 7β-hydroxy-*ent*-kaurenoic acid and GA_12_-aldehyde. Enzyme KAO is involved in this conversion (Figure 11). The first step involves stereospecific hydroxylation of C-7β. In the second reaction (initiated via stereospecific loss of the 6β-H), ring B contracts between 5 and 6 carbon atoms via migration of the C-7–C-8 bond from C-7 to C-6. The extrusion of C-7 as the aldehyde is observed. In the third step, GA_12_-aldehyde is oxidized to GA_12_. Cucurbitaceae plants, such as *Cucurbita maxima* and *Cucumis sativa*, contain GA_7_ oxidases (GA7ox) that can convert GA_12_-aldehyde into GA_12_. These enzymes are present in the development of seeds and vegetative tissues. The pathway branches from GA_12_. The reaction of 13-hydroxylation to GA_53_ initiates the formation of 13-hydroxylated GAs, such as GA_1_. Additionally, a parallel non-13-hydroxylation route from GA_12_ results in the synthesis of GA_4_ [158].

In higher plants, GA_12_ is present at a branch point along the GA pathway. GA_12_-aldehyde can be converted into GA_12_ or transformed into GA_53_ via the hydroxylation at C-13 (13-hydroxylated GA) (Figure 11). GA_12_ and GA_53_ are precursors of the non-13-hydroxylation and 13-hydroxylation pathways, respectively. These GAs can be oxidized at C-20, producing GA_9_ and GA_20_, respectively [159].

In rice, two cytochrome P450s—CYP714B1 and CYP714B2—are responsible for 13-hydroxylation of GA_12_. Although vegetative rice tissues contain predominantly 13-hydroxylated GAs, the overexpression of the CYP714B gene, which resulted in an increase in the concentration of various 13-hydroxy types of GAs, including GA_1_, also caused semidwarfism [173]. However, *A. thaliana* has two members of the CYP714A subfamily that are highly expressed in developing seeds. CYP714A1 transforms GA_12_ into 16α-carboxy-17-norGA_12_, whereas CYP714A2 converts GA_12_ into 12α-hydroxyGA_12_ (GA_111_) and, at a lower level of efficiency, GA_53_ via the 13-hydroxylation process. This enzyme is also capable of hydrolyzing *ent*-kaurenoic acid in the C-13 position to steviol [174]. Another member of the CYP714 family that is present in rice—CYP714D1—catalyzes the reaction of the epoxidation of the 16,17-double bond of 13-deoxyGAs, including GA_12_. This reaction leads to synthesis of inactive compounds [157]. Thus, members of the CYP714 family are responsible for the inactivation of GAs and *ent*-kaurenoids via their oxidation at the C- and D-rings. *A. thaliana* contains eight tandem CYP72A genes. Among these genes, CYP72A9 was shown to 13-hydroxylate GA_12_, as well as GA_9_ and GA_4_. Therefore, CYP72A9 is the main source of the 13-hydroxy types of Gas [175].

The transformation of GA_12_ and GA_53_ into GA_9_ and GA_20_, respectively, are catalyzed in plants by a family of 2-oxoglutarate/Fe(II)-dependent dioxygenases, which are also known as GA 20-oxidases (GA20ox) (Figure 11). These enzymes are responsible for the cleavage of C-20 with 19,10-γ-lactone formation, which is a characteristic of C_19_-GA. Firstly, C-20 methyl is oxidized into alcohol form. Next, it is transformed into the aldehyde. During this oxidation process, C-20 is removed as CO_2_, and 20-oic acid is a minor by-product. However, this biochemical mechanism is not well understood [176]. Vascular plants contain a family of *GA20ox* genes, which have different developmental, environmental, and tissue expression patterns. In many plants, GA20ox proteins are involved in the regulation of the GA rate of the synthesis of GA, and expressions of *GA20ox* genes are tightly regulated by developmental and environmental signals to maintain GA homeostasis [177].

In the final step in the biosynthesis of bioactive GAs, the C_19_-GAs (i.e., GA_9_ and GA_20_) are 3*β*-hydroxylated to GA_4_ and GA_1_, respectively, by the enzyme GA 3-oxidase (GA3ox) (Figure 11) [178]. GA_4_ is the first biologically active GA. This GA is desaturated to GA_7_, which is then converted into GA_3_ via the late 13-hydroxylation reaction. Enzymes GA3ox are highly active in vegetative tissues and function only as 3β-hydroxylases. They are characterized by high regiospecificity in dicots. On the other hand, GA3ox in monocots are less specific to the substrates. Therefore, GA_3_ is synthesized from GA20 as a minor by-product via the GA_1_ production route. The oxidation at the 2β and 3β positions leads to formation of the 2,3-unsaturated intermediate GA_5_, which is then converted into GA_3_ via oxidation at C-1β. This reaction is catalyzed by the same enzyme [157]. Therefore, monocots contain low levels of GA_3_, which are usually less than 10% of the GA_1_ content, while they are generally undetectable in vegetative tissues of dicots. In plants, the last step in GA synthesis is the 3β-hydroxylation of GA_9_ and GA_20_ into GA_4_ and GA_1_, respectively. GA_4_ and GA_1_ are the main GAs. The *GA3ox* genes are present in small families. *A. thaliana* contains four members, while rice and barley (*Hordeum vulgare*) contain only two members. Two enzymes of GA3ox—AtGA3ox1 and AtGA3ox2—present in *A. thaliana*, as well as only one enzyme found in cereals, are involved in the development of vegetative organs [178].

## 11. Strigolactones

Strigolactones (SLs), which are carotenoid-derived terpenoids, were initially characterized as germination stimulants for root parasitic plant witchweed (*Striga* spp.). SLs are a class of carotenoid-derived terpenoids that are involved in diverse developmental processes, such as seed germination, shoot branching, leaf senescence, and root development, as well as responses to various environmental conditions (e.g., light stress and high-temperature stress) [179]. They are also known as rhizosphere chemical signals that can regulate interactions between arbuscular mycorrhizal fungi and root parasitic plants. These groups of phytohormones also affect photosynthesis, synthesis, and action of other phytohormones, as well as regulating the levels of different metabolic compounds [180,181].

According to their chemical structures, SLs can be classified into two groups: canon-ical and non-canonical SLs. Canonical SLs contain a tricyclic lactone structure composed of three rings (ABC-rings) connected to a butenolide group (D-rings) through an enol-ether bridge, which is critical for the performance of their biological activities in plants. In contrast, non-canonical SLs do not have typical ABC-rings, though they contain both an enol-ether bridge and D-ring moieties [182].

**Figure 12:**
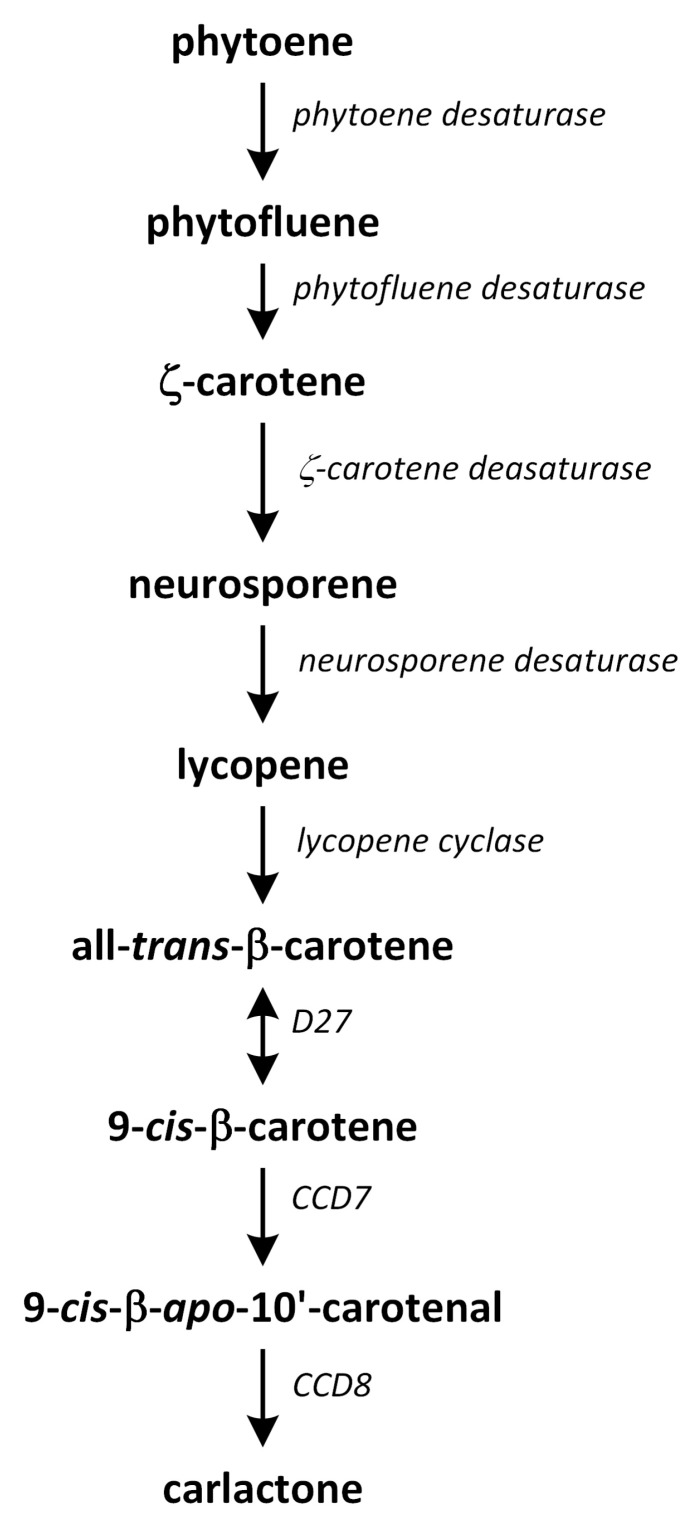
The biosynthetic pathways of carlactone.

Strigolactone biosynthesis mainly occurs in roots. The intermediates in the SL synthetic pathway have been also found in the stem. The biosynthesis of this group of phytohormones is tightly regulated by environmental conditions, such as starvation of phosphate or drought stress [183]. The early steps of SL biosynthesis are common for each type and may occur via the MVA or MEP pathways that lead to IPP synthesis, which is sequentially condensed to create DMAPP, geranyl pyrophosphate (GPP), farnesyl pyrophosphate (FPP), and GGPP (Figure 7) [116]. Thus, the first part of the SL biosynthetic pathway starts in chloroplasts. The condensation of two GGPP molecules by phytoene synthase generates phytoene (Figure 12), which is the first uncolored carotenoid [184]. Next, phytoene is transformed via a sequence of reactions of desaturation and *cis*/*trans*-isomerization into all-*trans*-lycopene. Cyclization reactions include conversion of all-*trans*-lycopene into all-*trans*-β-carotene and all-*trans*-α-carotene [185,186]. Dwarf27 (D27) isomerizes all-*trans*-β-carotene to 9-*cis*-β-carotene, followed by cleavage induced by carotenoid removal of enzyme dioxygenase 7 (CCD7) from 9-*cis*-β-apo-10′-carotenal, which undergoes cleavage and rearrangement reactions to create carlactone (CL), which is the central intermediate in SL biosynthesis (Figure 12). This reaction is catalyzed by the CCD8 enzyme [187]. CCD7 is encoded by gene *MAX3* and its orthologs genes: *RMS5* and *D17/HTD1*. Gene *MAX4* and its orthologs *RMS1*, *D10*, and Defective in Anther Dehiscence 1 (*DAD1*) encode CCD8. Both enzymes act in a progressive manner [188].

Carlactone (CL) contains only A- and D-rings that have an enol-ether bridge and exhibits SL-like properties. For example, CL inhibits shoot branching in SL biosynthetic mutants (i.e., rice, and *A. thaliana*) and promotes seed germination of *Striga hermonthica*. CL has been identified as an endogenous precursor in synthetic routes of both canonical and non-canonical SLs [186,189,190]. Furthermore, a new compound, which we identified as 3-hydroxyCL, is formed in vitro using 9-*cis*-3-hydroxy-β-apo-10′-carotenal in a reaction catalyzed by enzymes D27, CCD7, and CCD8. This compound was identified in plants such as rice, *A. thaliana*, and *N. benthamiana* [191].

**Figure 13:**
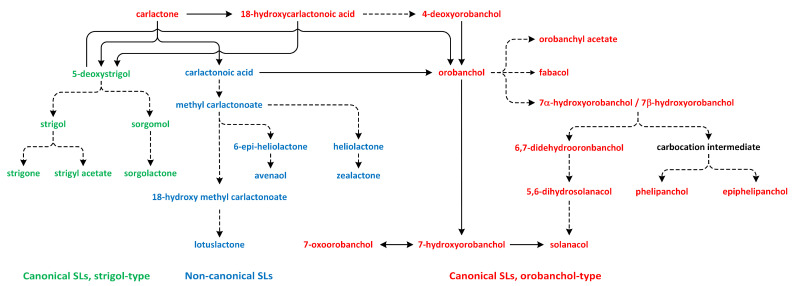
The biosynthetic pathways of different types of strigolactones (phytohormones are green, blue, and red).

The SL biosynthesis activates the biochemical conversions of CL through the participation of cytochrome P450 mono-oxygenases and other enzymes, which are responsible for the biological and structural diversity of these phytohormones. Subsequently, CL is transported from the chloroplast to the cytoplasm, where the reactions related to SL biosynthesis occur (Figure 13) [190].

The oxidation of CL results in the formation of carlactonoic acid (CLA) (Figure 13). This reaction is catalyzed by the More Axillary Growth 1 (MAX1) enzyme, which is a member of the CYP711A subfamily. The structure and biochemical properties of MAX1 have been well characterized. It is a conserved protein that possesses many homologs in a number of plant species. CLA can be treated as the precursor of strigol and orobanchol-type SL, i.e., 5-deoxystrigol (5DS) and 4-deoxyorobanchol (4DO). Enzymes identified in plants, such as Os900/OsCYP711A2 in rice (*Oryza sativa*), VuCYP722C in cowpea (*Vigna unguiculata*), SlCYP722C in tomato (*Solanum lycopersicum*), and GaCYP722C in cotton (*Gossypium arboreum*), take part in the synthesis of organic compounds 4-deoxyorobanchol, orobanchol, and 5-deoxystrigol, respectively, from CLA [189,190,192,193,194].

In the next reaction, CLA is transformed to methyl carlactonoate (MeCLA) by an unknown enzyme [189]. Next, Lateral Branching Oxidoreductase (LBO) converts methyl carlactonoate (MeCLA) into hydroxymethyl carlactonoate (1′-OH-MeCLA) and CLA [195]. LBO can produce CLA via direct demethylation of MeCLA or indirect spontaneous reversion of 1′-OH-MeCLA to CLA [196]. MeCLA is considered to be a precursor used for the synthesis of non-canonical SLs. Feeding experiments showed that MeCLA can be converted into heliolactone in sunflower and tobacco [193,194]. The enzyme responsible for the production of heliolactone has not yet been identified to confirm this theory, and it is also important to study the possibility that MeCLA is an important intermediate for the synthesis of other non-canonical SLs (e.g., avenaol and zealactone) (Figure 13) [196]. The enzymes that lay downstream and upstream of CL may be important for the production of various compounds that belong to the SL family. Carotenoid isomerase, CCD7, and CCD8 are able to transform all-*trans*-β-carotene into CL and convert 3-hydroxy-carlactone (3-OH-CL) through zeaxanthin [191]. Hydroxy-carlactone derivatives are the main SL in *A. thaliana* [195]. However, their roles in the regulation of plant growth and development have not yet been confirmed [196]. These non-canonical SLs are characterized by lower stability in relation to canonical SLs. Thus, studies of the biological functions of non-canonical SLs are difficult to carry out, and the results are incomplete [197].

## 12. Jasmonates

Jasmonates are plant hormones that originate from oxylipins involved in plant devel-opment and stress responses [198]. Their major representatives are the isomers of jasmonic acid (JA): (+)-7-iso-JA and (−)-JA. JA can be metabolized into methyl jasmonate (MeJA), which is a volatile phytohormone. Jasmonates have previously been defined as JA and its diverse metabolites derived from various reactions created via esterification, methylation, sulfation, glycosylation, conjugation, de-carboxylation, hydroxylation, and carboxylation [199]. Recently, an alternative and probably more ancient pathway was proposed. In this route, JA originates from dinor-OPDA (2,3-dinor-12-oxo-10,15(*Z*)-phytodienoic acid [dn-OPDA]) [200]. Thus, 12-oxo-10,15(*Z*)-phytodienoic acid (OPDA), dn-OPDA, and their derivatives are members of JAs family. JA and MeJA are the best characterized group of jasmonates in plants, and they are regarded as one of the major phytohormones that regulate both defense responses and the development process [201].

**Figure 14:**
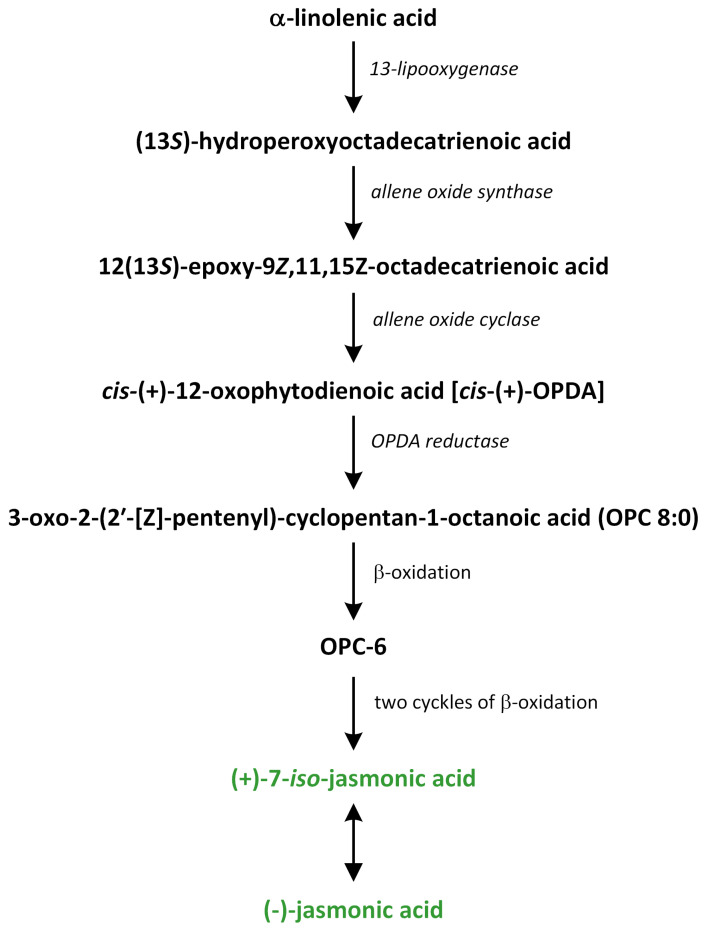
The biosynthetic pathways of jasmonic acid (phytohormones are green).

Jasmonate biosynthesis pathways have been extensively investigated in dicotyledonous plants, such as *A. thaliana*, tobacco, and tomato (Figure 14).

In monocotyledonous species, only a few biosynthetic enzymes of JA have been identified and characterized [202,203,204]. JA biosynthesis is initiated via the release of the polyunsaturated fatty acid α-linolenic acid 18:3 (α-LeA) from galactolipids, which are present in chloroplast membranes. This reaction of hydrolysis is catalyzed by phospholipase A_1_ (PLA_1_), which is characterized by specificity of sn-1 in the structure of fatty acids. Thus, α-LeA oxygenation is the first step in JA biosynthesis. The flower-specific protein DAD1, which is a PLA1, is required for the formation of JA [205,206].

The formation of JA occurs via one of the seven different branches of lipoxygenase (LOX) pathway [199]. Enzymes LOXs (lipoxygenases) are non-heme-iron-containing dioxygenases that produce hydroperoxides of fatty acid from polyunsaturated substrates. These enzymes can be sub-divided into 9-LOX and 13-LOX types, depending on the carbon atom to which molecular oxygen is added. In *A. thaliana*, the 13-LOX enzymes, such as AtLOX2, 3, 4, and 6, are pre-sent to the chloroplast. On the other hand, 9-LOX members (e.g., AtLOX1 and AtLOX5) are probably localized in the cytosol, although their functions are poorly understood [207,208]. The distribution and transport of α-LeA between the organelles, such as chloroplast and the cytosol, regulate the metabolic flow between 13-LOX- and 9-LOX-derived oxylipins [202].

In the chloroplast, (13*S*)-hydroperoxy octadecatrienoic acid (13-HPOT) is formed after oxygenation of *α*-LeA by the enzyme 13-LOX (Figure 14). This compound can play a role as a substrate of other enzymes, such as cytochrome P450 enzymes that belong to the CYP74 family, hydroperoxide lyase, allene oxide synthase (AOS), divinyl ether synthase, and epoxyalcohol synthase [209]. The next enzyme involved in the octadecanoid pathway is responsible for the biosynthesis of JA is AOS, which prefers to use 9- or 13-hydroperoxide derivatives as substrates. Thus, like LOX, this enzyme is characterized by 9- and 13-positional specificity. AOS is responsible for the formation of unstable allylic epoxides that can be spontaneously hydrolyzed in the presence of molecules of water in 13-hydroxy-12-oxo-octadecadienoic (α-ketols) and 9-hydroxy-12-oxo-octadecadienoic acids (γ-ketols). Moreover, the cyclization to a racemic mixture of *cis*-(+) and *cis*-(−) enantiomers is observed [210]. The *cis*-(+) isomer of oxylipin OPDA is produced in this reaction. This reaction requires the neighborhood of AOC. The stereochemistry of the pentenyl side chain and the carboxylic acid side chain at positions 3 and 7, respectively, of the pentanone ring in the JA structure is fixed in the step catalyzed via AOC and conserved in later reactions. In the cytosol, α-LeA can be oxygenated by enzyme 9-LOX and, subsequently, by enzyme 9-AOS to create two products: α-ketols and/or γ-ketols. On the other hand, α-LeA can also be oxygenated by α-dioxygenase (α-DOX) [211].

OPDA is known as a biosynthetic precursor of the phytohormone JA. It is also in-volved in various biological processes, which range from plant defence and stress responses to the regulation of growth and development [212]. OPDA is produced in the chloroplast through the 13-LOX/AOS pathway and then transported to peroxisomes. Recently, a plastid inner envelope-localized protein, known as OPDAT1, has been found in *Populus trichocarpa*. This observation suggested that this protein may participate in the export of OPDA from the chloroplast to other organelles [213]. OPDA can enter the peroxisome via diffusion of the protonated form of OPDA along its gradient. The cyclopentenone ring of OPDA after transportation into the peroxisome is reduced in the reaction catalyzed by the flavin-dependent oxidoreductase OPDA reductase 3 (OPR3). 3-oxo-2-(20-[*Z*]-pentenyl)-cyclopentane-1-octanoic acid (OPC-8) is produced during this step [214]. In the next reaction, OPC-8 is activated via esterification to CoA in the biochemical reaction catalyzed by OPC-8 coenzyme A ligase1, which is a member of the acyl-activating family of enzymes [215]. OPC-8 is then transformed into JA through three rounds of sequentially catalyzed peroxisomal β-oxidation reactions by acyl-CoA oxidase, which is a multifunctional protein, and L-3-ketoacyl-CoA thiolase [202]. During these reactions, six carbons are eliminated from the carboxy-terminal carbon chain. Therefore, the pentenyl side chain of JA is shortened in reactions that involve the ß-oxidation mechanism of the fatty acid [206].

Recently, an alternative pathway for JA synthesis has been proposed. Studies of the *opr3-3* mutant in which OPR3 activity is completely depleted showed that OPDA and hexadecatrienoic acid-derived dn-OPDA are metabolized to create tetranor-OPDA and 4,5-didehydro-JA. Next, this compound may be reduced to JA by OPR2 after being transported into the cytosol. This process suggests that the OPR2-dependent and OPR3-independent JA biosynthetic pathways may be very ancient in vascular plants and activated under different processes or the influence of certain environmental conditions [200,216,217].

## 13. Conclusions

In this review, the biosynthesis routes of all known plant phytohormones have been explored. Understanding these intricate biosynthetic pathways of plant hormones will provide valuable insights regarding their biological functions.

The biosynthesis of PAs begins with putrescine that leading to the production of other PAs. The ethylene synthesis pathway is more straightforward, with methionine being a precursor. Salicylic acid biosynthesis is believed to occur via two routes: the isochorismate synthase pathway and the phenylalanine ammonia-lyase pathway. Auxin synthesis in plants occurs via two main pathways: the Trp-dependent and independent routes. Four Trp-dependent IAA routes have been proposed: the IAM, IPA, TAM, and IAOX routes. Similarly, melatonin biosynthesis starts with Trp, which is synthesized de novo via the shikimate pathway. Two potential pathways of ABA biosynthesis have been identified in plants. The first pathway is direct, and the second pathway is indirect; in these pathways, ABA is derived from C_15_ farnesyl pyrophosphate and a C_40_ carotenoid, respectively. Brassinosteroid biosynthesis occurs through three pathways that lead to C_27_-, C_28_-, and C_29_-type BRs, with early steps happening via the MVA or MEP pathways. Cytokinins can be synthesized de novo as low-activity nucleotide mono-, di-, and tri-phosphates. Alternatively, cytokinins can be released from tRNA, creating nucleotide mono-phosphates, with the interconversions of nucleotides, nucleosides, and free bases catalyzed by enzymes involved in adenine metabolism. Gibberellins are formed from GGPP through IPP, which is the C-5 building block of all terpenoid/isoprenoid compounds, including BRs, CKs, ABA, and SLs. Carlactone is the main precursor of SL production in plants. Jasmonic acid synthesis begins with the release of polyunsaturated fatty acid α-linolenic acid from galactolipids present in chloroplast membranes.

The emergence of various phytohormone groups, along with their unique biosynthetic pathways, could be linked to their distinct chemical structures, biosynthetic path-ways, physicochemical properties, plant evolutionary histories, and roles in plants’ adaptation to diverse environmental conditions or developmental programs. Precursors or intermediates involved in one phytohormone pathway may also participate in the biosynthesis of another hormone, thus linking the pathways (Figure S1).

## Supplementary Materials

The following supporting information can be downloaded via the following link: https://www.mdpi.com/article/10.3390/metabo13080884/s1, Figure S1: Network of plant hormones’ biosynthetic pathways (all-in-one).

## Abbreviations

1′-OH-MeCLA	1′-hydroxymethyl carlactonoate
3-DT	3-dehydroteasterone
4DO	4-deoxyorobanchol
5DS	5-deoxystrigol
6-deoxo-3-DT	3-dehydro-6-deoxoteasterone
6-deoxoCT	6-deoxocathasterone
ABA	Abscisic acid
ACC	1-aminocyclopropane-1-carboxylic acid
ACO	ACC oxidase
ACS	ACC synthase
ADC	Aginine decarboxylase
Agm	Agmatine
AO1	Aldehyde oxidase 1
AOS	Allene oxide synthase
Arg	Arginine
ASMT	*N*-acetylserotonin *O*-methyltransferase
BL	Brassinolide
BR	Brassinosteroid
CCD7	Carotenoid removal dioxygenase 7
CK	Cytokinin
CL	Carlactone
CLA	Carlactonoic acid
CN	Campestanol
COMT	Caffeic acid *O*-methyltransferase
CPP	*Ent*-copalyl diphosphate
CPS	*Ent*-copalyl diphosphate synthase
CR	Cholesterol
CS	Castasterone
CT	Cathasterone
*c*Z	*Cis*-zeatin
DAD1	Defective in Anther Dehiscence 1
dcSAM	Decarboxylated S-adenosyl-L-methionine
DL	Dolicholide
DMAPP	Dimethylallyl pyrophosphate
dn-OPDA	2,3-dinor-12-oxo-10,15(*Z*)-phytodienoic acid
DS	Dolichosterone
DOXP	1-deoxy-D-xylulose-5-phosphate
DXS	DOXP synthase
FPP	Farnesyl pyrohosphate
GA	Gibberellin
GGPP	Geranylgeranyl pyrophosphate
GGPPS	Geranylgeranyl pyrophosphate synthase
GPP	Geranyl pyrophosphate
HMBDP	*E*-4-hydroxy-3-methyl-but-2-enyl diphosphate
IAA	Indole-3-acetic acid
IAM	Indole-3-acetamide
IAN	Indole-3-acetonitrile
IAOX	Indole-3-acetaldoxime
ICS	Isochorismate synthase
ICS-Glu	Isochorismate-9-glutamate
iP	*N*^6^-∆^2^-isopentenyl adenine
IPA	Indole-3-pyruvic acid
IPP	Isopentenyl diphosphate
JA	Jasmonic acid
KAO	*Ent*-kaurenoic acid oxidase
KO	*Ent*-kaurene oxidase
KS	*Ent*-kaurene synthase
LBO	Lateral Branching Oxidoreductase
MAX1	More Axillary Growth 1
MeJA	Methyl jasmonate
MEP	Methylerythritol phosphate
MTA	5′-methylthioadenosine
MVA	Mevalonate
NCPA	*N*-carbamoyl putrescine
ODC	Ornithine decarboxylase
OPC-8	3-oxo-2-(20-[*Z*]-pentenyl)-cyclopentane-1-octanoic acid
OPDA	12-oxo-*Z*-phytodienoic acid
OPR3	Oxidoreductase OPDA reductase 3
Orn	Ornithine
PA	Polyamine
PAL	Phenylalanine ammonia-lyase
PLP	Pyridoxal-5′-phosphate
Put	Putrescine
SA	Salicylic acid
SAM	S-adenosyl-L-methionine
SAMDC	S-adenosyl-L-methionine decarboxylases
SL	Strigolactone
SMO1	C-4 sterol methyl oxidase 1
SNAT	Serotonin *N*-acetyltransferase
Spd	Spermidine
Spm	Spermine
SPMS	Spermine synthase
T5H	Tryptamine 5-hydroxylase
TAM	Tryptamine
TDC	Tryptophan decarboxylase
TPH	Tryptophan hydroxylase
Trp	Tryptophan
TY	Typhasterol
*t*Z	*Trans*-zeatin
α-LeA	α-linolenic acid 18:3
