# Supplementary material for: Biosynthetic Pathways of Hormones in Plants
Source: Metabolites. 2023 Jul 25;13(8):884. doi: 10.3390/metabo13080884 (PMC10456939; doi:10.3390/metabo13080884)
Supplement: Supplementary file 1 [file metabolites-13-00884-s001.zip › metabolites-2518633-supplementary latest.pdf]

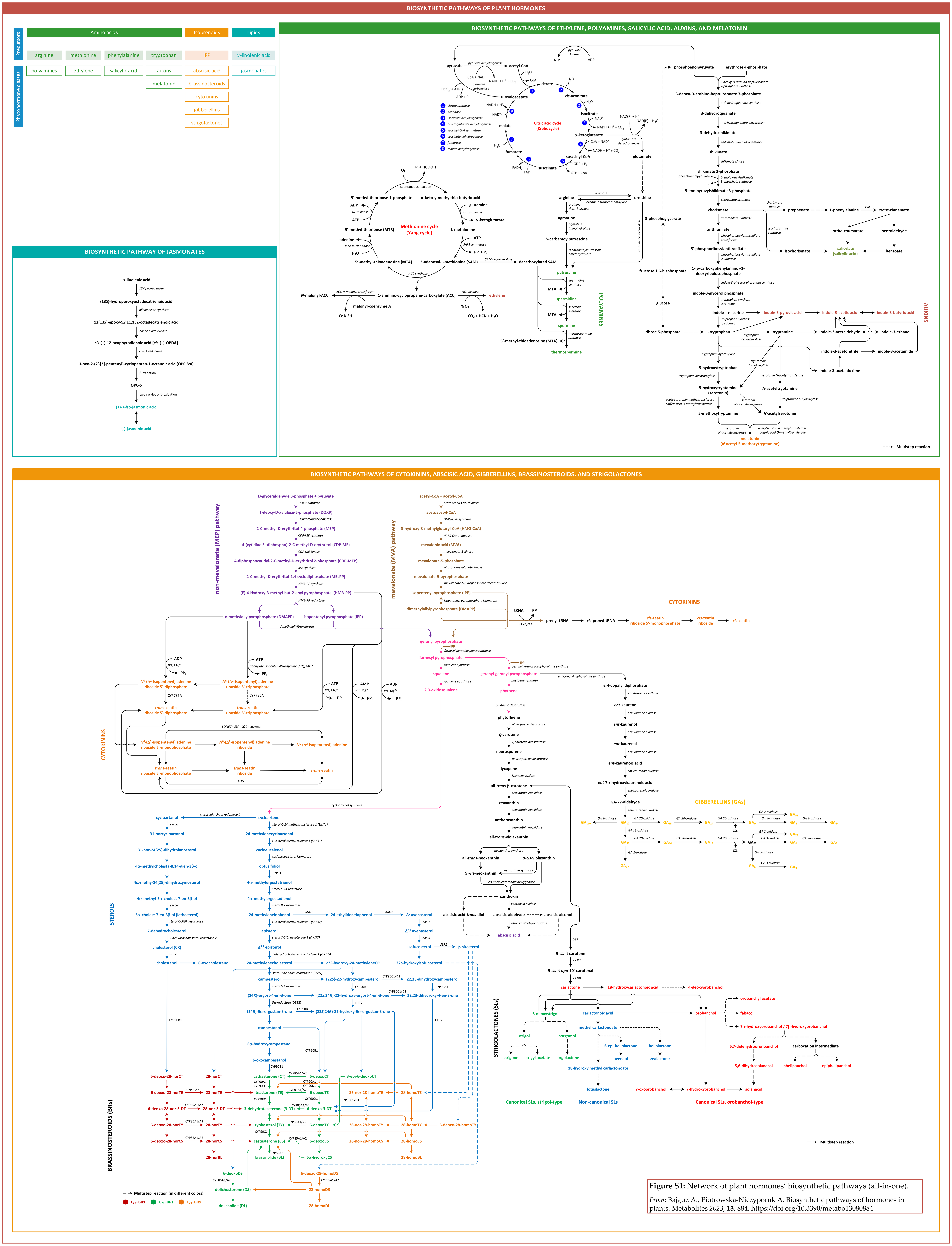

Figure S1: Network of plant hormones' biosynthetic pathways (all-in-one).

From: Bajguz A., Piotrowska-Niczyporuk A. Biosynthetic pathways of hormones in plants. Metabolites *2023*, **13**, 884. https://doi.org/10.3390/metabo13080884
